# Tuning Anisotropic Optical Properties of Inorganic and Hybrid Organic–Inorganic MXenes via Topochemical Surface Modification

**DOI:** 10.1021/acs.nanolett.6c03430

**Published:** 2026-07-22

**Authors:** Young-Hwan Kim, Hui Fang, Hanaul Noh, Abu Rashed Md. Shawon, Sanguk Han, Daniel Intriago, Teng Zhang, Ashley E. Cavanagh, Gusaimas M. H. H. Akbar, Francisco Lagunas, Yury Gogotsi, Aleksandra Vojvodic, Aaron J. Rossini, Zahra Fakhraai, Dmitri V. Talapin

**Affiliations:** † Pritzker School of Molecular Engineering, 2462 University of Chicago, Chicago, Illinois 60637, United States; ‡ Department of Chemistry, 6572 University of Pennsylvania, Philadelphia, Pennsylvania 19104, United States; § 531012 Park Systems Inc., Santa Clara, California 95054, United States; ∥ Department of Chemistry, 1177 Iowa State University, Ames, Iowa 50011, United States; ⊥ Division of Materials Science and Engineering, Ames National Laboratory, Ames, Iowa 50011, United States; # Department of Chemical and Biomolecular Engineering, 6572 University of Pennsylvania, Philadelphia, Pennsylvania 19104, United States; 7 Department of Materials Science and Engineering and A.J. Drexel Nanomaterials Institute, 6527 Drexel University, Philadelphia, Pennsylvania 19104, United States; 8 Department of Mechanical Engineering and Materials Science, Washington University in St. Louis, St. Louis, Missouri 63130, United States; 9 Institute for Material Science and Engineering, Washington University in St. Louis, St. Louis, Missouri 63130, United States; 10 Department of Chemistry and James Franck Institute, 2462 University of Chicago, Chicago, Illinois 60637, United States; 11 Center for Nanoscale Materials, 1291 Argonne National Laboratory, Lemont, Illinois 60439, United States

**Keywords:** MXenes, Topochemical synthesis, Optical properties, Hybrid materials, Surface functionalization

## Abstract

Surface groups are central to the properties of MXenes, yet their role in optical anisotropy remains largely unexplored. Here, we use a topochemical route to synthesize single crystals of stacked Ti_3_C_2_Cl_2_ and hybrid organic–inorganic MXenes (*h*-MXenes) with lateral sizes of 38–75 μm, rotational registry, and tunable interlayer spacing. Solid-state NMR spectroscopy shows that topochemical substitution generates mixed amido, imido, and hydride surface motifs, which modify the electronic structure of the Ti_3_C_2_ inorganic core. Imaging spectroscopic ellipsometry with micron-scale spatial resolution enables reconstruction of the complex dielectric tensor of individual multilayer crystals. Ti_3_C_2_Cl_2_ exhibits a type-II hyperbolicity above 930 nm, whereas *h*-MXenes do not display hyperbolicity within the measured 300–1700 nm window, instead showing reduced in-plane conductivity, suppressed out-of-plane light absorption, and a chain-length-dependent blue shift of a near-infrared absorption feature. These results demonstrate topochemical surface modification as a direct handle for engineering MXenes as surface-programmable optical media.

## Graphical abstract

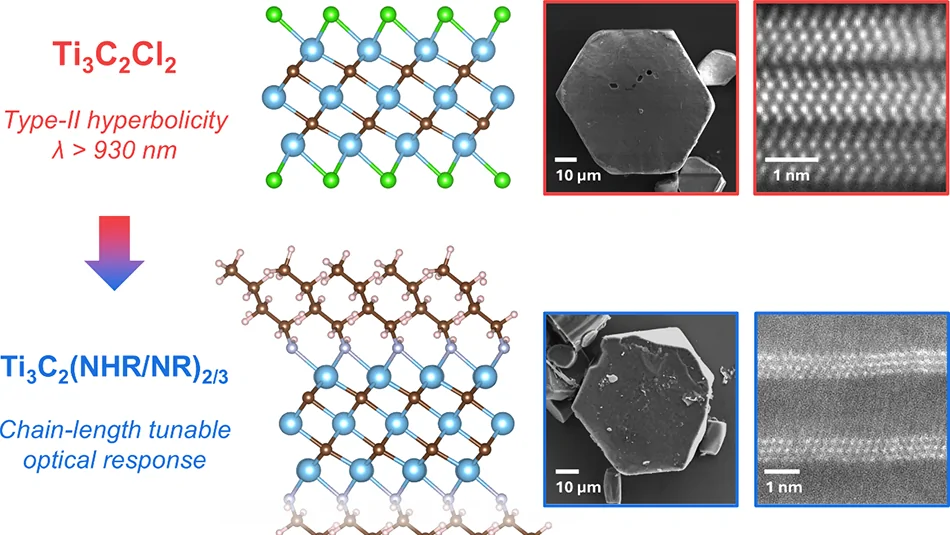

MXenes are a class of two-dimensional (2D) transition-metal carbides and nitrides, whose combination of metallic conductivity, structural anisotropy, and chemically addressable surfaces has opened opportunities across energy storage, catalysis, electronics, and photonic technologies.^1–3^ In contrast to many other 2D materials, their properties are not defined solely by the 2D crystal structure, but also by the chemistry of surface terminations, which directly modulates charge density, interlayer interactions, and electronic structure.^4,5^ This coupling between a conductive transition-metal carbide/nitride core and a highly tunable surface makes MXenes a particularly attractive platform for chemically controlling functional properties.

Recent advances in postsynthetic covalent surface modification have shown that halogen-terminated MXenes can serve as versatile synthetic platforms for installing new functional groups while preserving the inorganic core.^6–10^ A particularly important development has been the emergence of hybrid organic–inorganic MXenes (*h*-MXenes), where halogen-terminated Ti_3_C_2_Cl_2_ undergoes ligand exchange with primary alkylamines to form alkylamido/imido-functionalized derivatives.^11^ These materials retain the layered Ti_3_C_2_ framework while all halides are replaced with alkylamido/imido groups, allowing systematic control over the interlayer spacing through the choice of organic ligand.

Such surface modification is especially compelling for investigating anisotropic optical response, as it can alter both the properties of Ti_3_C_2_ sheets, such as band structure and carrier concentration, and the dielectric environment between adjacent layers. However, although anisotropic charge transport and magnetic properties in MXenes have been reported,^12–14^ their anisotropic optical properties remain largely unexplored. Although optical anisotropy in MXene sheets has been predicted through density functional theory (DFT),^15–17^ direct experimental determination of the dielectric tensor has remained challenging.

This challenge stems from the intrinsic thinness of the MXene flakes, which complicates edge-on normal incidence reflectometry, as with other 2D materials.^18^ Consequently, previous ellipsometry studies on MXenes have largely relied on isotropic models for data fitting.^12,19–25^ In these ensemble films, stacking, overlap, and inhomogeneous flake orientation create averaging effects that blur the distinction between in-plane and out-of-plane optical behaviors. Therefore, evaluating optical anisotropy and its evolution with surface chemistry requires direct experimental determination of the directional optical constants in well-aligned systems.

Here, we combine topochemical synthesis with imaging spectroscopic ellipsometry (SE) to resolve the anisotropic optical response and reconstruct the dielectric tensor of single-crystalline Ti_3_C_2_Cl_2_ and *h*-MXenes across the ultraviolet–near-infrared (UV–NIR) range. Figure 1 illustrates the transformation of the Ti_3_AlC_2_ MAX phase into Ti_3_C_2_Cl_2_ MXene, followed by surface ligand exchange resulting in *h*-MXenes. The Ti_3_AlC_2_ precursor is converted to Ti_3_C_2_Cl_2_ via reaction with molten CdCl_2_ at elevated temperatures,^8^ after which Ti_3_C_2_Cl_2_ undergoes covalent surface modification with deprotonated primary alkylamines to yield *h*-MXenes bearing amido/imido ligands (Figure 1a).^11^ Here, we note that surface refers to the basal-plane surface of each Ti_3_C_2_ layer, and the covalent modification extends across the entire bulk multilayer stack rather than only at the exposed outermost interface.

**Figure 1:**
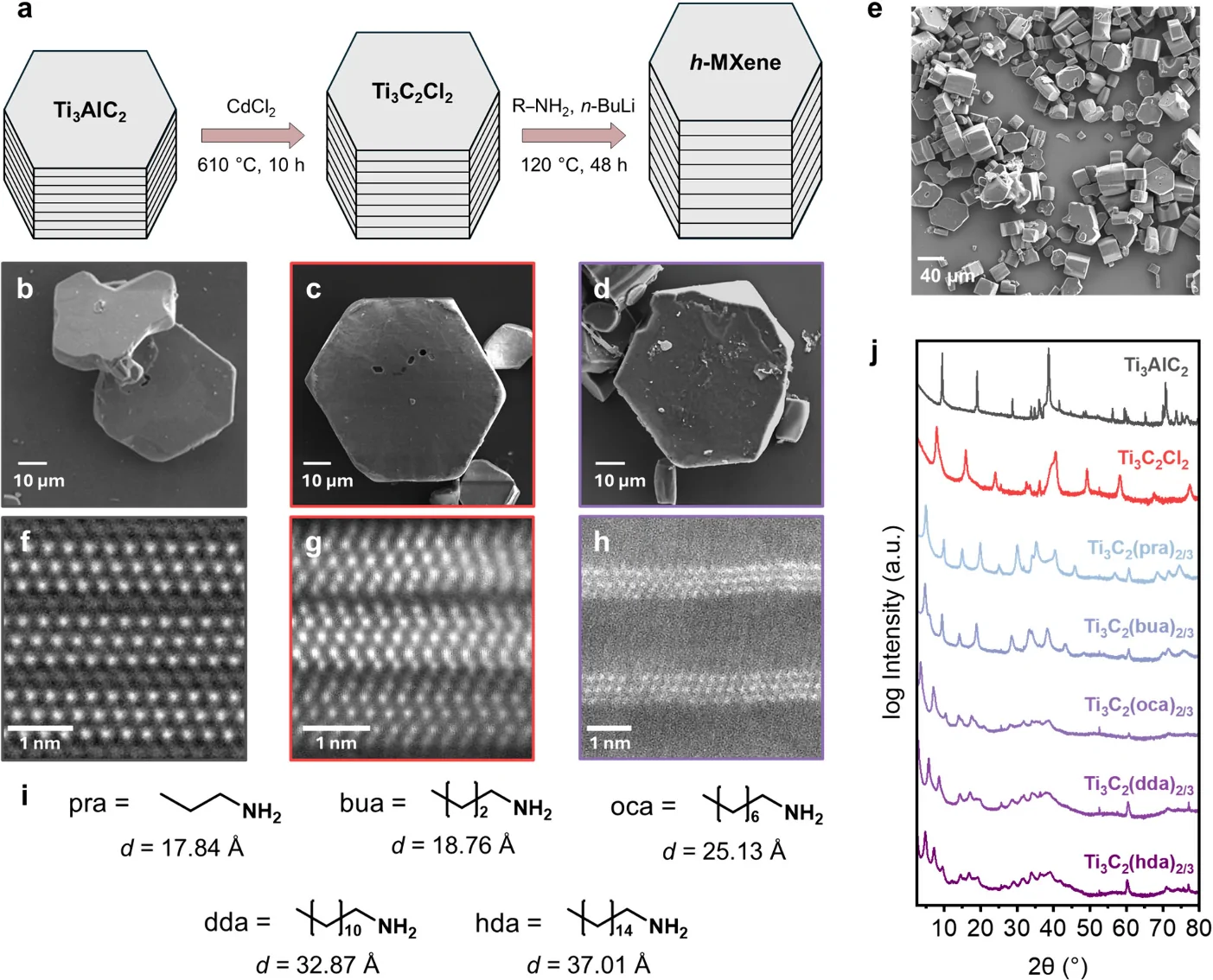
Topochemical transformation from Ti_3_AlC_2_MAX phase to Ti_3_C_2_Cl_2_ and *h*-MXene crystals. (a) Schematic illustration of the topochemical conversion of single-crystalline grains of Ti_3_AlC_2_ MAX phase to Ti_3_C_2_Cl_2_ and *h*-MXenes. (b–d) SEM images of Ti_3_AlC_2_ MAX phase, Ti_3_C_2_Cl_2_, and Ti_3_C_2_(dda)_2/3_ *h*-MXene, showing preservation of the macroscopic hexagonal particle morphology and size. (e) A low-magnification SEM image of Ti_3_C_2_(hda)_2/3_ *h*-MXene particles. (f–h) STEM cross-sectional images of Ti_3_AlC_2_, Ti_3_C_2_Cl_2_, and Ti_3_C_2_(dda)_2/3_ *h*-MXene, showing topochemical expansion of interlayer spacing upon etching and ligand exchange. (i) Selection of primary alkylamine ligands used to synthesize *h*-MXenes with tunable interlayer spacing. (j) PXRD patterns of Ti_3_AlC_2_, Ti_3_C_2_Cl_2_, and *h*-MXenes functionalized with different surface alkylamido/imido ligands. Patterns are displayed on a logarithmic scale to show in-plane diffraction features.

Notably, this entire sequence proceeds topochemically, preserving both the macroscopic particle morphology and the microscopic crystal structure. The Ti_3_AlC_2_ MAX phase synthesized under aluminum-rich conditions exhibits single-crystalline hexagonal particles with lateral size of 38–75 μm,^26–28^ as shown by scanning electron microscopy (SEM, Figure 1b). These particles can be topochemically transformed into Ti_3_C_2_Cl_2_ MXene (Figure 1c) and subsequently into a series of *h*-MXenes (Figure 1d), maintaining their hexagonal morphology and lateral size without delamination or fragmentation of Ti_3_C_2_ sheets. The ligands we employed here include *n*-propylamine (pra), *n*-butylamine (bua), *n*-octylamine (oca), *n*-dodecylamine (dda), and *n*-hexadecylamine (hda). The low-magnification SEM image of Ti_3_C_2_(hda)_2/3_ *h*-MXene particles (Figure 1e) further confirms that this morphological and size preservation persists across the entire particle population and highlights the anisotropic plate-like shape of the synthesized MXenes. Scanning transmission electron microscopy (STEM) reveals topochemical expansion of the interlayer spacing upon etching and ligand exchange, while preserving the *P*6_3_ */mmc* space group of the Ti_3_C_2_ sublattice characteristic of the parent structure (Figure 1f–h).

Because the ligand exchange proceeds topotactically, the interlayer spacing of the resulting MXenes can be systematically tuned by varying the alkyl-chain length of the primary amine used for substitution (Figure 1i). Powder X-ray diffraction (PXRD) confirms the linear relationship between the interlayer spacing and the alkyl chain length of the amine ligands employed (Figure 1j, Figure S1). SEM-energy dispersive spectroscopy (SEM-EDS) confirms the removal of surface Cl terminations after the reaction (Figures S2 and S3).

Magic angle spinning (MAS) solid-state NMR (ssNMR) spectroscopy is a powerful probe of the molecular-level structure of MXenes and related two-dimensional compounds.^10,11^ To establish the surface chemistry generated by topochemical amine substitution, Ti_3_C_2_Cl_2_ was reacted with 20% ^15^N isotope-enriched butylamine (bua). The ^1^H-detected ^1^H­{^15^N} cross-polarization (CP) heteronuclear correlation (HETCOR) NMR spectrum reveals two distinct nitrogen environments, assigned to surface alkylamido (−NHR; – 22 ppm) and alkylimido (=NR; 35 ppm) motifs (Figure 2a), consistent with our previous studies.^11^ The dependence of the ^15^N NMR signal intensities on CP contact time further supports this assignment. At a short contact time (0.5 ms), the ^15^N NMR spectra obtained by summing the columns of the HETCOR NMR spectra are dominated by the amido resonance, whereas the acquisition with a longer contact time (3.0 ms) reveals the imido resonance (Figure 2b). Fitting the peaks with Voigt functions in the summed ^15^N NMR spectra extracted from the HETCOR spectrum that was acquired with the longer 3.0 ms contact time gives an apparent amido:imido ratio of 48:52.

**Figure 2:**
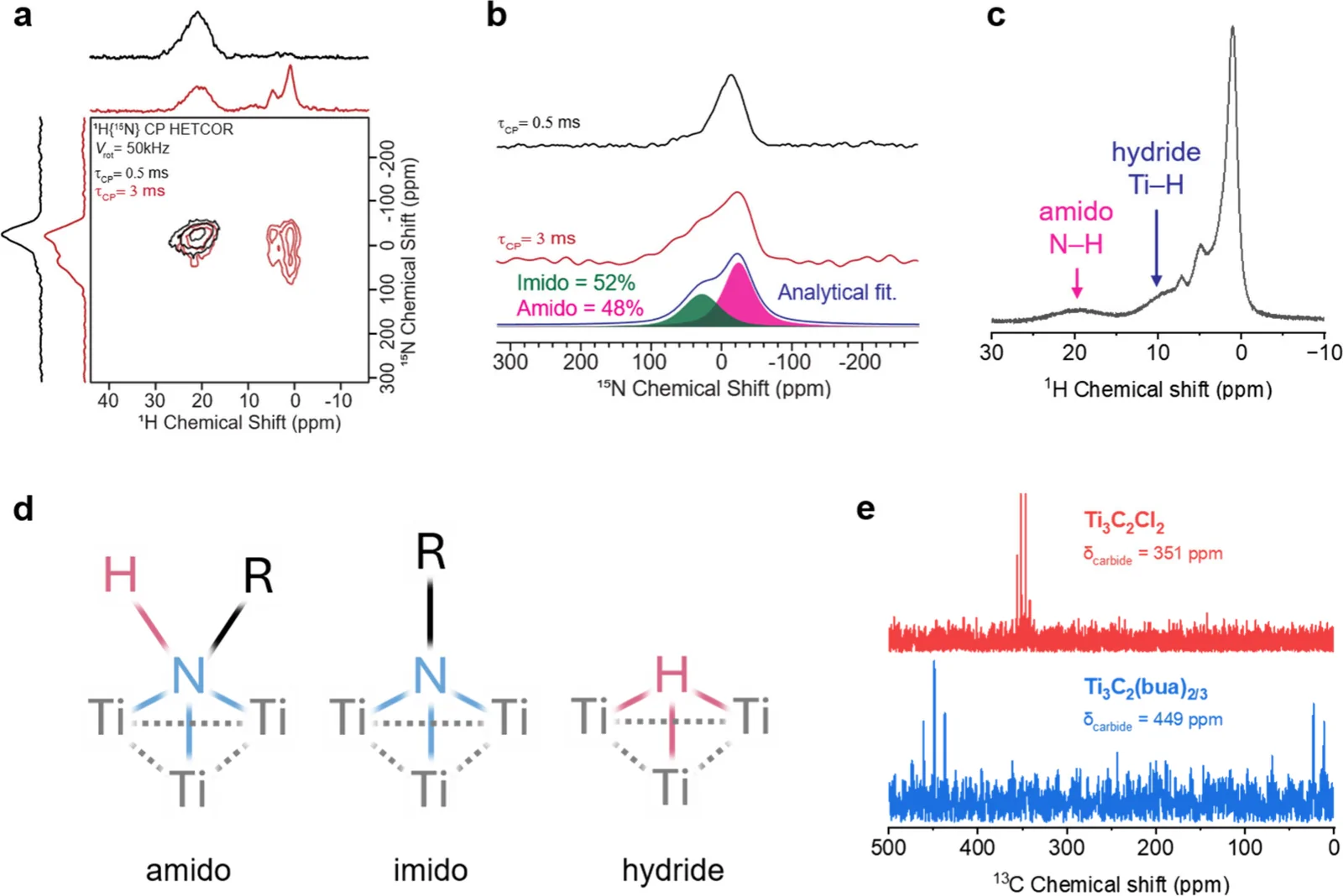
MAS solid-state NMR spectroscopy analyses of Ti_3_C_2_Cl_2_ and ^15^N-enriched Ti_3_C_2_(bua)_2/3_MXenes. (a) ^1^H­{^15^N} CP-HETCOR NMR spectra of Ti_3_C_2_(bua)_2/3_ recorded with a short back CP contact time of 0.5 ms (black), and a longer back CP contact time of 3.0 ms (red). (b) ^15^N ssNMR spectra of Ti_3_C_2_(bua)_2/3_ extracted from the sum of the ^1^H­{^15^N} CP-HETCOR spectra. Fits of the amido and imido peaks to Voigt functions with relative peak areas are shown. (c) ^1^H spin echo NMR spectra of Ti_3_C_2_(bua)_2/3_. (d) Hypothesized surface bonding motifs on *h*-MXenes. Each motif corresponds to alkylamido (−NHR), alkylimido (=NR), and hydride (−H), respectively. (e) ^13^C CPMG NMR spectra of Ti_3_C_2_Cl_2_ and Ti_3_C_2_(bua)_2/3_, showing distinct δ_carbide_. All NMR experiments were performed with a 50 kHz MAS frequency.

^1^H spin–echo ssNMR spectra show alkyl-chain resonances near 1 ppm and a strongly deshielded resonance at 20 ppm, assigned to amido N–H protons (Figure 2c). In addition, a broad shoulder centered near 9 ppm is observed, which we previously attributed to surface hydride species.^11^ Taken together, the ^1^H and ^15^N ssNMR data indicate that the surface of amine-based *h*-MXenes is a mixture of alkylamido, alkylimido, and hydride motifs (Figure 2d).

Beyond identifying surface ligands, ssNMR spectroscopy also provides a probe of the inorganic core. Especially in metallic MXenes, the ^13^C carbide resonance is known to be highly sensitive to the electronic environment of the metal carbide sheets, which has been attributed to the Knight shift contribution from the conduction electrons.^29–31^ To determine whether amine substitution perturbs the electronic structure of the Ti_3_C_2_ core, we acquired ^13^C Carr–Purcell–Meiboom–Gill (CPMG) ssNMR spectra of Ti_3_C_2_Cl_2_ and Ti_3_C_2_(bua)_2/3_ (Figure 2e). Both materials exhibit carbide resonances in an unusually deshielded region that is far outside the typical range of diamagnetic organic ^13^C chemical shift ranges, suggesting that the individual Ti_3_C_2_ sheets retain a metallic character. Importantly, the resonance position is strongly dependent on the surface termination, where Ti_3_C_2_Cl_2_ shows δ_carbide_ = 351 ppm, whereas Ti_3_C_2_(bua)_2/3_ exhibits a markedly shifted resonance at δ_carbide_ = 449 ppm. As established in previous studies,^29–31^ this pronounced termination-dependent ^13^C chemical shift suggests that covalent surface modification changes the electronic structure of the Ti_3_C_2_ carbide core. Consistent with this interpretation, the band structure of Ti_3_C_2_Cl_2_ and Ti_3_C_2_(bua)_2/3_ obtained with DFT calculations shows that replacement of chloride by alkylamido/imido surface groups modifies the electronic structure of the MXene sheets (Figure S4). The pulse sequences used for the ssNMR experiments are shown in Figure S5.

The topochemical synthesis yields high-quality single crystals with lateral dimensions of ∼75 μm, enabling imaging SE on individual MXene stacks. Conventional SE of 2D materials is typically performed on flakes or films, where the relatively large probe spot primarily probes in-plane optical responses. In contrast, imaging SE with a reduced spot size (∼1 μm), applied to stacked single-crystalline particles, provides direct sensitivity to both in-plane and out-of-plane light-matter interactions, enabling reconstruction of the full complex dielectric tensor and a rigorous assessment of optical anisotropy.^32^

Imaging SE measurements were performed at room temperature on single crystals of Ti_3_C_2_Cl_2_ and *h*-MXenes. For each material, measurements were collected with three distinct configurations to enable reconstruction of the full permittivity tensor (Figure 3). An edge-on particle oriented vertically on the substrate was measured in the (90°, 90°, 0°) and (0°, 90°, 0°) configurations, where the (90°, 90°, 0°) configuration was acquired first, followed by a 90° rotation of the substrate to obtain the (0°, 90°, 0°) configuration (Figures 3b,c). A face-on particle lying horizontally on the substrate from the same batch was subsequently measured in the (0°, 0°, 0°) configuration (Figure 3d). Here, the measurement configurations are defined by the Euler angles (φ, θ, ψ).

**Figure 3:**
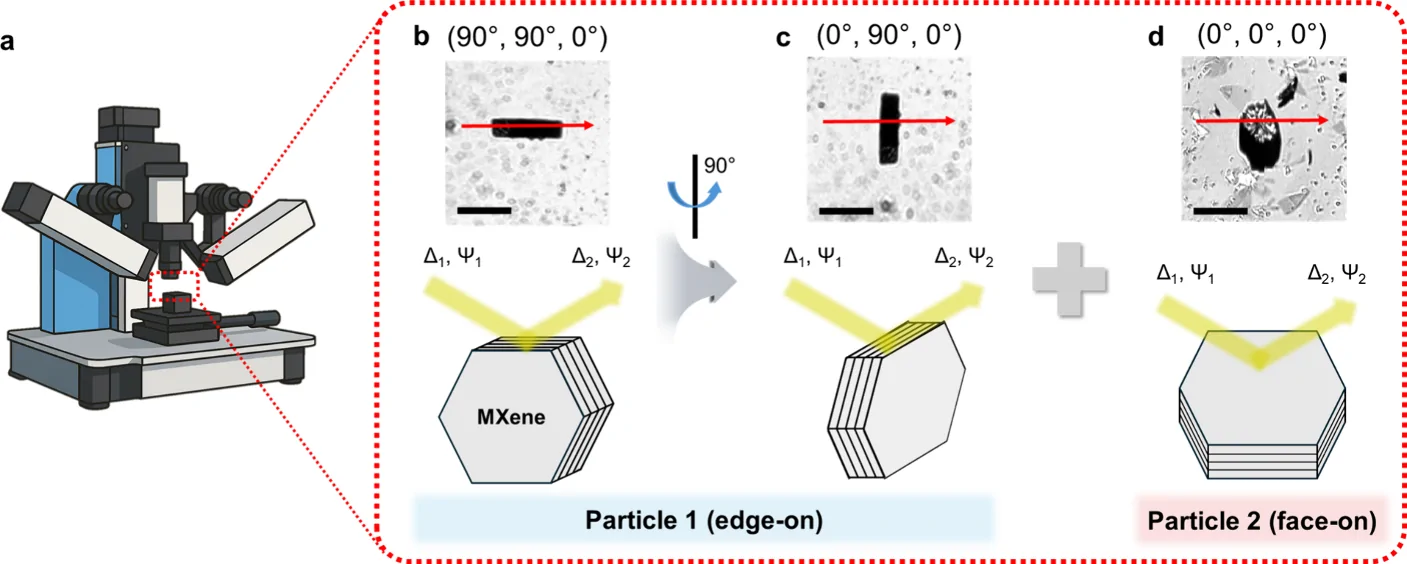
Imaging spectroscopic ellipsometry measurement configurations definition. (a) Schematic of the imaging ellipsometer setup, integrating a spectroscopic ellipsometer with an optical microscope to probe single particles. (b–d) Schematic and optical micrographs of MXene crystallites used for the measurement. Measurements were performed in three distinct orientations to enable reconstruction of the full permittivity tensor: (90°, 90°, 0°), (0°, 90°, 0°), and (0°, 0°, 0°). The optical micrograph was acquired directly using the imaging ellipsometer during the measurement. Scale bar, 100 μm.

The complex dielectric constants were extracted by fitting the SE results to a uniaxial anisotropic model, where the ordinary axis corresponds to the symmetric in-plane dielectric response (ε_//_) of the MXene lattice, while the extraordinary axis describes the out-of-plane response (ε_⊥_) perpendicular to the basal plane. The fitting model was composed of multiple harmonic oscillators and/or a Drude oscillator in each direction. Figure S6 presents the measured and fitted SE angles Ψ­(λ) and Δ­(λ) of Ti_3_C_2_Cl_2_ and *h*-MXenes, obtained from a global fit combining the (90°, 90°, 0°) and (0°, 90°, 0°) configurations. Measurements acquired in the (0°, 0°, 0°) configuration were used to cross-validate the extracted dielectric functions; in this geometry, an isotropic model incorporating only the in-plane dielectric constants was sufficient to fit the experimental data in this geometry. Additional details of the SE fitting are provided in the Supporting Information, and the results are tabulated in Tables S1–S9.

Figure 4 presents the SE-measured real (ε_1_) and imaginary (ε_2_) parts of the dielectric constants as a function of wavelength (Figures 4b–e) for Ti_3_C_2_Cl_2_ and Ti_3_C_2_(bua)_2/3_ MXenes, with their corresponding atomic structures shown in Figure 4a. The oscillators to construct ε_2_ are shown in Figures S7 and S8, and the corresponding real and imaginary components of the complex refractive index (*n*, *k*) are provided in Figures S9 and S10.

**Figure 4:**
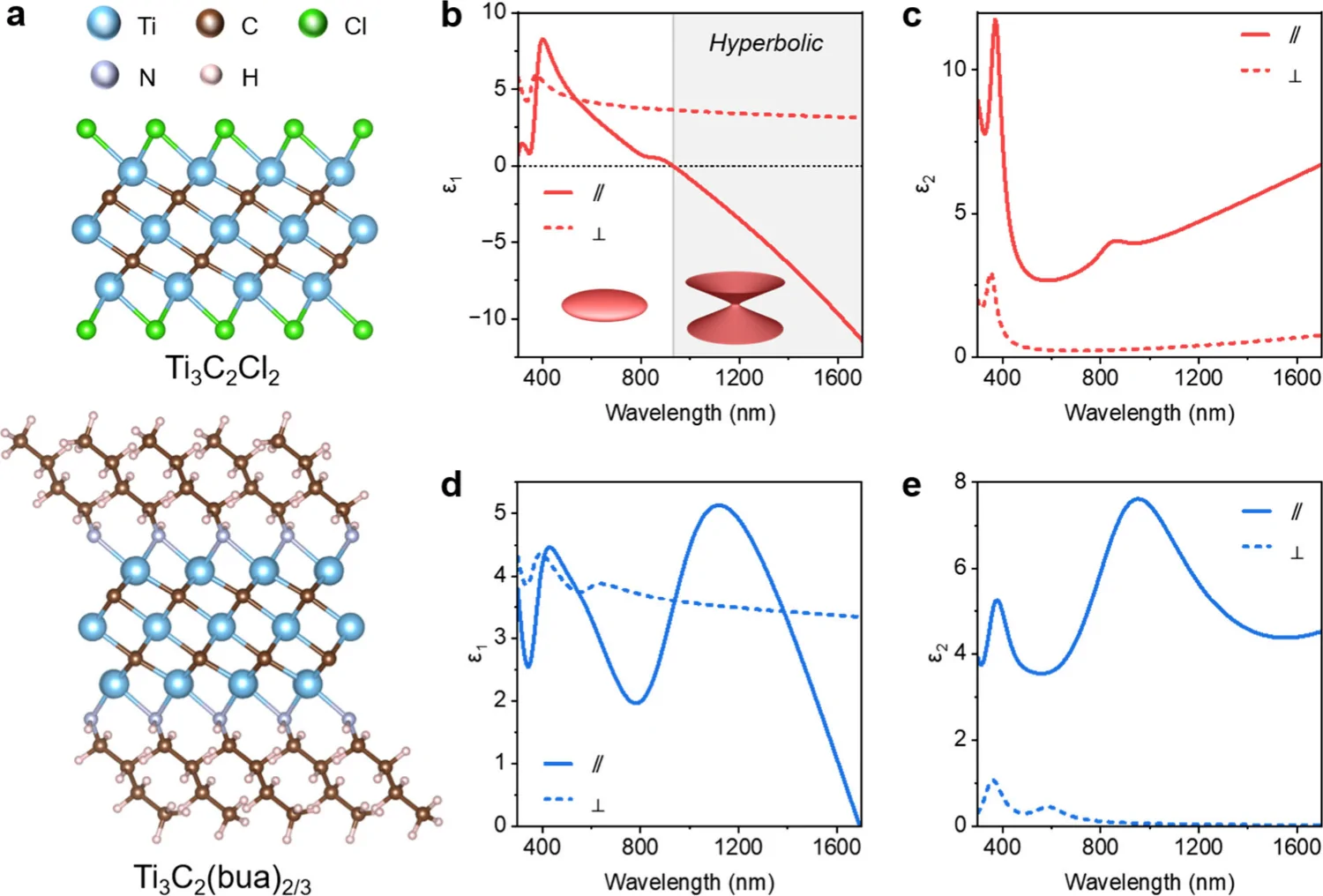
SE-measured complex dielectric function of Ti_3_C_2_Cl_2_ and Ti_3_C_2_(bua)_2/3_ MXene. (a) DFT-relaxed atomic structure of Ti_3_C_2_Cl_2_ and Ti_3_C_2_(bua)_2/3_. (b, c) Real (ε_1_) and imaginary (ε_2_) parts of the dielectric constant as a function of wavelength for Ti_3_C_2_Cl_2_. The inset of (b) shows the isofrequency contours calculated at 900 nm (left) and 1000 nm (right) from the dielectric functions in (b, c). // and ⊥ denote the in-plane and out-of-plane components. The gray region in panel (b), where ε_//_ < 0 and ε_⊥_ > 0, indicates the hyperbolic regime of Ti_3_C_2_Cl_2_. (d, e) Real (ε_1_) and imaginary (ε_2_) parts of the dielectric constant as a function of wavelength for Ti_3_C_2_(bua)_2/3_.

Ti_3_C_2_Cl_2_ shows a type-II hyperbolic regime that emerges above the epsilon-near-zero (ENZ) point at 930 nm (1.33 eV), where the in-plane ε_1_ becomes negative (ε_//_ < 0) while the out-of-plane ε_1_ remains positive (ε_⊥_ > 0) (Figure 4b,c). Hyperbolic dispersion has been realized primarily in artificially engineered metamaterials,^33,34^ and more recently in several van der Waals materials and their heterostructures,^35–38^ which motivates the search for materials that intrinsically exhibit hyperbolic response.^34,35,38–42^ In such media, the resulting hyperbolic isofrequency contours (IFCs) are open in momentum space, enabling propagating modes with large wavevectors and a correspondingly enhanced photonic density of states. These properties have enabled a range of nanophotonic applications, including subdiffraction imaging,^43^ enhanced spontaneous emission (Purcell effect),^44^ and negative refraction.^45^

While theoretical studies have suggested that MXenes may exhibit intrinsic dielectric anisotropy that could give rise to hyperbolic dispersion,^15,46^ the present measurements provide direct experimental verification of this anisotropy and resulting hyperbolic response. The IFCs calculated from the extracted dielectric function show a topological transition from an ellipsoidal dispersion at 900 nm to a hyperbolic dispersion at 1000 nm (Figure 4b, Figure S11). The hyperbolic quality factor, defined as |ε_//_/ε_⊥_|, reaches 1.7 at 1700 nm (Figure S12).^35^ This behavior is consistent with our first-principles calculations, where DFT followed by perturbation optical response calculations reproduce the ENZ point at 913 nm (1.36 eV) and the hyperbolic regime at longer wavelengths (Figure S13). Details on first-principles calculations are provided in the Supporting Information.

The negative ε_1_ below the plasma frequency arises from the polarization response of free electrons in the opposite direction to the electric field (Figure 4b). The increase of ε_2_ as the wavelength increases above 1000 nm for measurements made both in-plane and out-of-plane in Ti_3_C_2_Cl_2_ MXenes (Figure 4c) is due to absorption by free electrons. This portion of the spectrum can be fitted to the Drude function to extract the resistivity (Figure S7). The resistivity of of in-plane (0.0014 ± 0.0001 Ω·cm) is 5 times lower than that out-of-plane (0.008 ± 0.002 Ω·cm), indicating significant anisotropy of Ti_3_C_2_Cl_2_ stack, which is less conductive and more transparent out-of-plane. This trend is consistent with the previously measured and calculated results on randomly stacked MXene flakes,^12,47^ and the measured in-plane resistivity values are also comparable with the previous results measured for monoflake Ti_3_C_2_Cl_2_ MXenes.^19^

In addition to the Drude conductivity, a high-energy (<600 nm) extinction peak is observed in both in-plane and out-of-plane ε_2,_ which can be assigned to an interband transition (IBT). The small-amplitude peak around 850 nm (∼1.47 eV) is only observed on the in-plane ε_2_. A similar extinction peak was reported for monoflake Ti_3_C_2_Cl_2_ MXenes and attributed to an interband transition.^19^ Our DFT-calculated joint density of states (JDOS) further supports this assignment (Figure S14). Figure 4d,e presents the real (ε_1_) and imaginary (ε_2_) parts of the dielectric constant as a function of wavelength for Ti_3_C_2_(bua)_2/3_ MXene. In contrast to Ti_3_C_2_Cl_2_, no negative ε_1_ is observed for Ti_3_C_2_(bua)_2/3_ MXene within the measurable spectral range (300–1700 nm) (Figure 4d). This suggests that the hyperbolic region of *h*-MXenes, if present, is shifted to a lower energy beyond the detection limit (>1700 nm) upon surface substitution. Additionally, Ti_3_C_2_(bua)_2/3_ MXenes exhibit a pronounced broad peak in ε_2_ around 1000 nm (Figure 4e), indicating strong absorption in the NIR region, which can be attributed to an IBT. The NIR tail of the in-plane ε_2_ spectra for Ti_3_C_2_(bua)_2/3_ can be fitted using either a harmonic or Drude oscillator (Figure S15). Due to the limited spectral window, distinguishing between the two fitting approaches remains challenging. Assuming a Drude model, an in-plane resistivity (0.0035 ± 0.0006 Ω·cm) can be deduced, which is 2.5 times higher than that of Ti_3_C_2_Cl_2_. This suggests that organic ligands are reducing the in-plane electrical conductivity. No peak is observed in the NIR region of the out-of-plane ε_2_ spectra for Ti_3_C_2_(bua)_2/3_, indicating negligible conductivity and increased optical transparency in this direction. This can be attributed to the presence of the dielectric organic layers, which act as barriers hindering polarization coupling and electron transport across the interlayer space. These bulky ligands increase the spacing between the conductive MXene layers, thus increasing the hopping distance and lowering the probability of electrons being transported between the layers.

Figure 5 shows the combination of dielectric constant spectra of *h*-MXenes with different alkylamine ligands ranging from propylamine to hexadecylamine, shown schematically in Figure 1i. The corresponding refractive index spectra is shown in Figure S16. We note that the fit uncertainty increases for the longer-chain members (Tables S8 and S9), likely reflecting the greater conformational disorder of the longer alkyl chains, which can broaden and weaken the spectral features that constrain the anisotropic fit.

**Figure 5:**
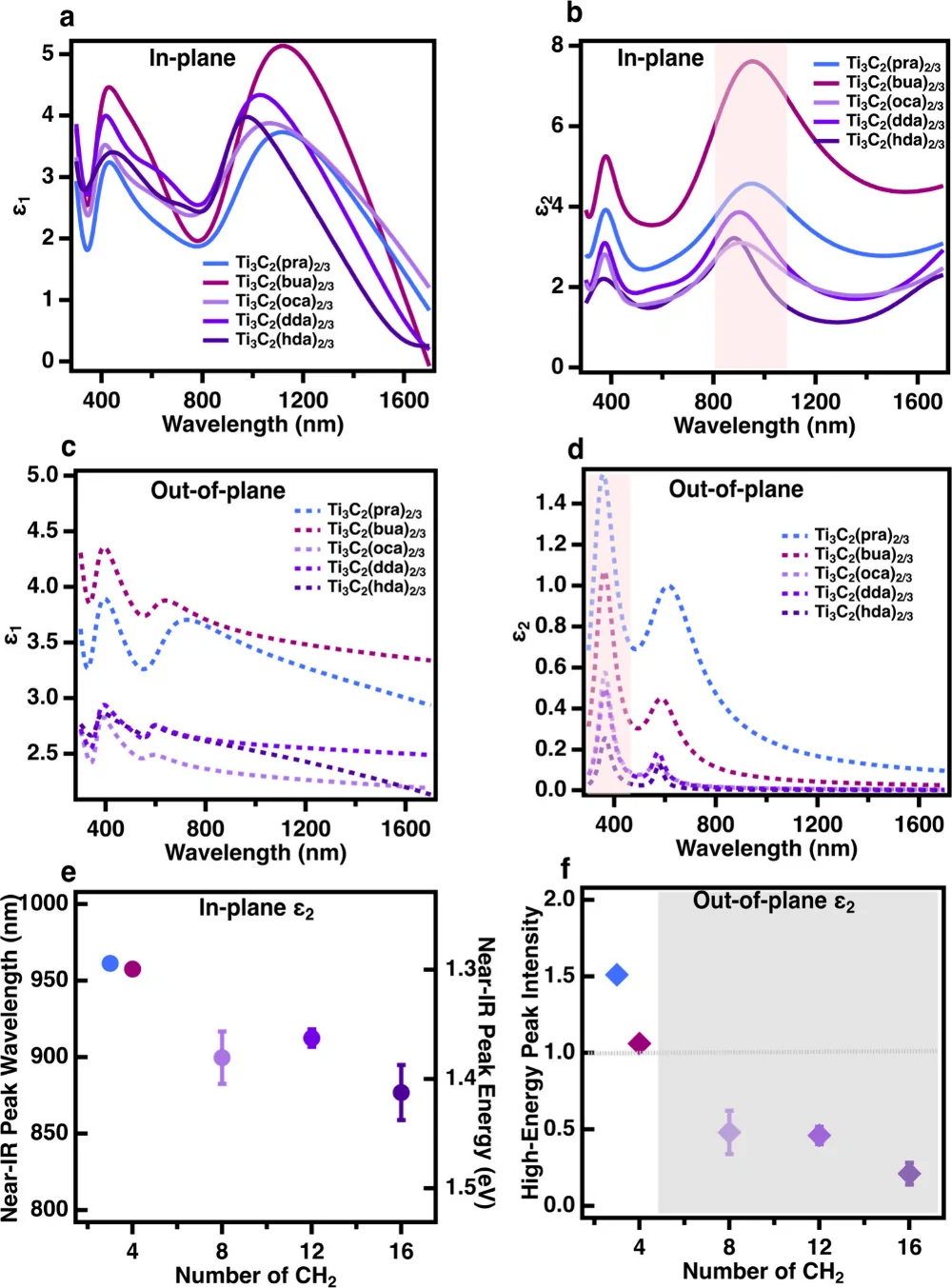
Complex dielectric function of different *h*-MXenes. (a, b) The in-plane real (ε_1_) and imaginary (ε_2_) parts of the dielectric constants as a function of wavelength for *h*-MXenes. (c, d) The out-of-plane real (ε_1_) and imaginary (ε_2_) parts of the dielectric constants as a function of wavelength for *h*-MXenes. (e) Changes of the in-plane ε_2_ near-IR peak (pink highlight area in panel b) wavelength (or energy) versus number of carbon atoms in the interlayer alkyl groups. (f) Changes in the out-of-plane ε_2_ high-energy peak (pink highlight area in panel d) intensity as a function of carbon atoms in the interlayer alkyl groups. An intensity less than 1 indicates low absorption, which can be assumed to be a transparent layer.

No hyperbolic behavior is observed within the measurable spectral range for any of the *h*-MXenes (Figure 5a). Notably, the NIR peak in the in-plane ε_2_ shifts to higher energy as the length of organic ligands increases (Figure 5b,e). This trend is also visible in the raw SE data Ψ (Figure S17), confirming that it does not originate from fitting artifacts. The observed blue shift is inconsistent with a surface plasmon resonance (SPR), which would typically red-shift with increasing dielectric separation.^48^ Instead, the trend suggests that the feature originates from an interband transition, whose energy is modified by changes in the dielectric environment introduced by the organic layers. Similar shifts induced by dielectric screening of interband transitions have been reported in other low-dimensional materials, such as quantum dots or 2D semiconductors.^49,50^

For the out-of-plane ε_2_, the intensity of the two IBT peaks decreases progressively with increasing ligand lengths (Figure 5d). In particular, the higher-energy peak diminishes from ∼1.5 for Ti_3_C_2_(pra)_2/3_ to below 1 for Ti_3_C_2_(oca)_2/3_ and further to ∼0.2 for Ti_3_C_2_(hda)_2/3_ (Figure 5f). An intensity below 1 indicates negligible absorption across the measured spectral window. This behavior suggests that the material behaves as an optically transparent medium in the out-of-plane direction. Consistent with this interpretation, the out-of-plane dielectric response of Ti_3_C_2_(oca)_2/3_ can be equally well fitted using an alternative Cauchy transparent-layer model (Figure S18c,d), yielding results comparable to those obtained with a low-absorption oscillator model. In contrast, the Cauchy transparent-layer model fails to reproduce the Ti_3_C_2_(bua)_2/3_ data (Figure S18a,b), as its ε_2_ intensity remains above 1 (Figure 5f), indicating substantial absorption and the absence of transparency in that system.

This chain-length-dependent out-of-plane transparency can be reproduced by effective medium theory analysis (Figure S19),^51^ suggesting that it arises from the classical electromagnetic averaging. In contrast, the NIR peak shift in the in-plane ε_2_ spectra (Figure 5b,e) is not captured by this model (Figure S20), indicating that changes in the intrinsic optical response of the Ti_3_C_2_ layers may vary across the *h*-MXene series, possibly due to differences in surface-motif distribution.

In summary, we demonstrate topochemical surface modification as a chemically precise route to tune the anisotropic optical response of multilayer MXenes. By preserving single-particle morphology from Ti_3_AlC_2_ to Ti_3_C_2_Cl_2_ and subsequently to covalently modified *h*-MXenes, this synthesis yields crystals suitable for imaging spectroscopic ellipsometry and direct reconstruction of the full dielectric tensor. Ti_3_C_2_Cl_2_ exhibits pronounced optical anisotropy, including a directly observed type-II hyperbolic regime above 930 nm. In contrast, this hyperbolicity is suppressed by surface substitution with alkylamido/imido motifs. The resulting *h*-MXenes instead show a chain-length-dependent blue shift of the in-plane NIR peak and a progressive decrease in the out-of-plane absorption feature, demonstrating that surface chemistry provides an effective handle for modulating their optical response. These findings establish MXenes as an intrinsically hyperbolic medium and a chemically tunable platform for anisotropic optical functionality, expanding prospects for their use in advanced photonic and optoelectronic applications.

## Supplementary Material



## Notes

Supporting Information: Materials and Methods, supporting figures and tables, including additional structural characterization, DFT-calculated electronic structures, ssNMR pulse sequences, and spectroscopic ellipsometry fitting results (PDF)

## References

[1] VahidMohammadi A., Rosen J., Gogotsi Y. (2021). The World of Two-Dimensional Carbides
and Nitrides (MXenes). Science.

[2] Anasori B., Lukatskaya M. R., Gogotsi Y. (2017). 2D Metal Carbides and Nitrides (MXenes)
for Energy Storage. Nat. Rev. Mater..

[3] Zhang D., Shah D., Boltasseva A., Gogotsi Y. (2022). MXenes for Photonics. ACS Photonics.

[4] Jiang M., Wang D., Kim Y.-H., Duan C., Talapin D. V., Zhou C. (2024). Evolution of Surface Chemistry in Two-Dimensional MXenes: From Mixed
to Tunable Uniform Terminations. Angew. Chem.,
Int. Ed..

[5] Zhou J., Dahlqvist M., Björk J., Rosen J. (2023). Atomic Scale Design
of MXenes and Their Parent Materials–From Theoretical and Experimental
Perspectives. Chem. Rev..

[6] Li M., Lu J., Luo K., Li Y., Chang K., Chen K., Zhou J., Rosen J., Hultman L., Eklund P., Persson P. O. Å., Du S., Chai Z., Huang Z., Huang Q. (2019). Element Replacement
Approach by Reaction with Lewis Acidic Molten
Salts to Synthesize Nanolaminated MAX Phases and MXenes. J. Am. Chem. Soc..

[7] Li Y., Shao H., Lin Z., Lu J., Liu L., Duployer B., Persson P. O. Å., Eklund P., Hultman L., Li M., Chen K., Zha X.-H., Du S., Rozier P., Chai Z., Raymundo-Piñero E., Taberna P.-L., Simon P., Huang Q. (2020). A General Lewis Acidic Etching Route
for Preparing MXenes with Enhanced Electrochemical Performance in
Non-Aqueous Electrolyte. Nat. Mater..

[8] Kamysbayev V., Filatov A. S., Hu H., Rui X., Lagunas F., Wang D., Klie R. F., Talapin D. V. (2020). Covalent Surface
Modifications and Superconductivity of Two-Dimensional Metal Carbide
MXenes. Science.

[9] Ding H., Li Y., Li M., Chen K., Liang K., Chen G., Lu J., Palisaitis J., Persson P. O. Å., Eklund P., Hultman L., Du S., Chai Z., Gogotsi Y., Huang Q. (2023). Chemical Scissor–Mediated
Structural Editing of Layered Transition Metal Carbides. Science.

[10] Zhou C., Kim Y.-H., Atterberry B., Khokhar V., Thind A. S., Lagunas F., Lin R., Wang D., Cho W., Zhou Z., Czaikowski M. E., Filatov A. S., Anderson J. S., Klie R. F., Schaller R. D., Jiang D., Rossini A. J., Talapin D. V. (2025). MXenoids: Generalization
of MXene-Inspired Covalent
Surface Modifications Across Two-Dimensional Materials. J. Am. Chem. Soc..

[11] Zhou C., Wang D., Lagunas F., Atterberry B., Lei M., Hu H., Zhou Z., Filatov A. S., Jiang D., Rossini A. J., Klie R. F., Talapin D. V. (2023). Hybrid Organic–Inorganic
Two-Dimensional Metal Carbide MXenes with Amido- and Imido-Terminated
Surfaces. Nat. Chem..

[12] Fang H., Fang Z., Thakur A., Anasori B., Rappe A. M., Fakhraai Z. (2025). Different Charge Transport
Mechanisms in Ti3C2Tx MXene
Monoflakes and Multiflakes. J. Phys. Chem. Lett..

[13] de
Leuze O., Fernandes F. M., Arib S., Caputo L., Fontes A. P., Nguyen V.-H., Pazniak H., Nysten B., Charlier J.-C., Hermans S., Hackens B. (2025). Anisotropic Charge
Transport in 2D Single Crystals of Ti_3_C_2_T_x_ MXenes. Commun. Mater..

[14] Frey N. C., Bandyopadhyay A., Kumar H., Anasori B., Gogotsi Y., Shenoy V. B. (2019). Surface-Engineered
MXenes: Electric Field Control of
Magnetism and Enhanced Magnetic Anisotropy. ACS Nano.

[15] Lashgari H., Abolhassani M. R., Boochani A., Elahi S. M., Khodadadi J. (2014). Electronic
and Optical Properties of 2D Graphene-like Compounds Titanium Carbides
and Nitrides: DFT Calculations. Solid State
Commun..

[16] Xu M., Yang J., Liu L. (2019). Temperature-Dependent Optical and
Electrical Properties of Bulk Ti_2_AlC and Two-Dimensional
MXenes from First-Principles. Phys. B Condens.
Matter.

[17] Bai Y., Zhou K., Srikanth N., Pang J. H. L., He X., Wang R. (2016). Dependence
of Elastic and Optical Properties on Surface Terminated
Groups in Two-Dimensional MXene Monolayers: A First-Principles Study. RSC Adv..

[18] Ruta F. L., Sternbach A. J., Dieng A. B., McLeod A. S., Basov D. N. (2020). Quantitative
Nanoinfrared Spectroscopy of Anisotropic van Der Waals Materials. Nano Lett..

[19] Fang H., Fang Z., Thakur A., Rad V., Chandran
Balachandran Sajitha N., Michałowski P.
P., Soroush M., Anasori B., Rappe A. M., Fakhraai Z. (2026). Band-like Optical Signatures
of Ti_3_C_2_T_x_ MXenes. J. Phys. Chem. C.

[20] Shamsabadi A. A., Fang H., Zhang D., Thakur A., Chen C. Y., Zhang A., Wang H., Anasori B., Soroush M., Gogotsi Y., Fakhraai Z. (2023). The Evolution
of MXenes Conductivity
and Optical Properties Upon Heating in Air. Small Methods.

[21] Fang H., Thakur A., Zahmatkeshsaredorahi A., Fang Z., Rad V., Shamsabadi A. A., Pereyra C., Soroush M., Rappe A. M., Xu X. G., Anasori B., Fakhraai Z. (2024). Stabilizing
Ti_3_C_2_T_
*x*
_ MXene Flakes
in Air by Removing Confined Water. Proc. Natl.
Acad. Sci. U. S. A..

[22] Chaudhuri K., Alhabeb M., Wang Z., Shalaev V. M., Gogotsi Y., Boltasseva A. (2018). Highly Broadband
Absorber Using Plasmonic Titanium
Carbide (MXene). ACS Photonics.

[23] Dillon A. D., Ghidiu M. J., Krick A. L., Griggs J., May S. J., Gogotsi Y., Barsoum M. W., Fafarman A. T. (2016). Highly Conductive
Optical Quality Solution-Processed Films of 2D Titanium Carbide. Adv. Funct. Mater..

[24] Furchner A., Parker T., Mauchamp V., Hurand S., Plaickner J., Rappich J., Emerenciano A. A., Hinrichs K., Gogotsi Y., Petit T. (2025). Ti_3_C_2_T*
_x_
* MXene Thin
Films and Intercalated Species Characterized by IR-to-UV Broadband
Ellipsometry. J. Phys. Chem. C.

[25] Liu Z., El-Demellawi J. K., Bakr O. M., Ooi B. S., Alshareef H. N. (2022). Plasmonic
Nb_2_CT_x_ MXene-MAPbI_3_ Heterostructure
for Self-Powered Visible-NIR Photodiodes. ACS
Nano.

[26] Mathis T. S., Maleski K., Goad A., Sarycheva A., Anayee M., Foucher A. C., Hantanasirisakul K., Shuck C. E., Stach E. A., Gogotsi Y. (2021). Modified MAX Phase
Synthesis for Environmentally Stable and Highly Conductive Ti_3_C_2_ MXene. ACS Nano.

[27] Michałowski P. P., Anayee M., Mathis T. S., Kozdra S., Wójcik A., Hantanasirisakul K., Jóźwik I., Piątkowska A., Możdżonek M., Malinowska A., Diduszko R., Wierzbicka E., Gogotsi Y. (2022). Oxycarbide MXenes and
MAX Phases Identification Using Monoatomic Layer-by-Layer Analysis
with Ultralow-Energy Secondary-Ion Mass Spectrometry. Nat. Nanotechnol..

[28] Anayee M., Wang R. J., Downes M., Ippolito S., Gogotsi Y. (2025). Layer-by-Layer
Mechanism of the MAX Phase to MXene Transformation. Matter.

[29] Brette F., Kourati D., Paris M., Loupias L., Célérier S., Cabioc’h T., Deschamps M., Boucher F., Mauchamp V. (2023). Assessing
the Surface Chemistry of 2D Transition Metal Carbides (MXenes): A
Combined Experimental/Theoretical ^13^C Solid State NMR Approach. J. Am. Chem. Soc..

[30] Griffith K. J., Hope M. A., Reeves P. J., Anayee M., Gogotsi Y., Grey C. P. (2020). Bulk and Surface
Chemistry of the Niobium MAX and MXene
Phases from Multinuclear Solid-State NMR Spectroscopy. J. Am. Chem. Soc..

[31] Hope M. A., Forse A. C., Griffith K. J., Lukatskaya M. R., Ghidiu M., Gogotsi Y., Grey C. P. (2016). NMR Reveals
the
Surface Functionalisation of Ti_3_C_2_ MXene. Phys. Chem. Chem. Phys..

[32] Choi B., Lynch J., Chen W., Jeon S.-J., Cho H., Yang K., Kim J., Engheta N., Jariwala D. (2026). Natural Hyperbolicity
of Hexagonal Boron Nitride in the Deep Ultraviolet. Nat. Commun..

[33] Poddubny A., Iorsh I., Belov P., Kivshar Y. (2013). Hyperbolic Metamaterials. Nat.
Photonics.

[34] Lee D., So S., Hu G., Kim M., Badloe T., Cho H., Kim J., Kim H., Qiu C.-W., Rho J. (2022). Hyperbolic
Metamaterials:
Fusing Artificial Structures to Natural 2D Materials. eLight.

[35] Korzeb K., Gajc M., Pawlak D. A. (2015). Compendium of Natural Hyperbolic
Materials. Opt. Express.

[36] Lee M., Lee E., So S., Byun S., Son J., Ge B., Lee H., Park H. S., Shim W., Pee J. H., Min B., Cho S.-P., Shi Z., Noh T. W., Rho J., Kim J.-Y., Chung I. (2021). Bulk Metamaterials Exhibiting Chemically
Tunable Hyperbolic Responses. J. Am. Chem. Soc..

[37] Lynch J., Peng T.-Y., Yang J.-W., Conran B. R., Choi B., Chen C. Y., Fakhraai Z., McAleese C., Lu Y.-J., Jariwala D. (2025). High-Temperature-Resilient Hyperbolicity in a Mixed-Dimensional
Superlattice. Matter.

[38] Ruta F. L., Shao Y., Acharya S., Mu A., Jo N. H., Ryu S. H., Balatsky D., Su Y., Pashov D., Kim B. S. Y., Katsnelson M. I., Analytis J. G., Rotenberg E., Millis A. J., van Schilfgaarde M., Basov D. N. (2025). Good Plasmons in
a Bad Metal. Science.

[39] Venturi G., Mancini A., Melchioni N., Chiodini S., Ambrosio A. (2024). Visible-Frequency
Hyperbolic Plasmon Polaritons in a Natural van Der Waals Crystal. Nat. Commun..

[40] Li Y., Zhang Y., Zhang W., Li X., Tang J., Xiao J., Zhang G., Liao X., Jiang P., Liu Q., Luo Y., Cao Z., Lyu Q., Tong Y., Yang R., Yang H., Sun Q., Gao Y., Wang P., Chen Z., Liu W., Wang S., Lyu G., Hu X., Aeschlimann M., Gong Q. (2025). Broadband Near-Infrared
Hyperbolic Polaritons in MoOCl_2_. Nat. Commun..

[41] Ermolaev G., Toksumakov A., Slavich A., Minnekhanov A., Tselikov G., Mazitov A., Kruglov I., Tikhonowski G., Mironov M., Radko I. P., Grudinin D., Fradkin I., Vyshnevyy A., Sofer Z., Arsenin A., Novoselov K. S., Volkov V. (2026). Giant Optical Anisotropy and Visible-Frequency Epsilon-near-Zero
in Hyperbolic van der Waals MoOCl_2_. Nano Lett..

[42] Ghosh A., Raab C., Spellberg J. L., Mohan A., Munawar M., Rieger J., King S. B. (2026). Spatiotemporal
Visualization of Long-Range
Anisotropic Plasmon Polaritons in Hyperbolic MoOCl_2_. Nat. Commun..

[43] Liu Z., Lee H., Xiong Y., Sun C., Zhang X. (2007). Far-Field
Optical Hyperlens
Magnifying Sub-Diffraction-Limited Objects. Science.

[44] Krishnamoorthy H. N. S., Jacob Z., Narimanov E., Kretzschmar I., Menon V. M. (2012). Topological Transitions in Metamaterials. Science.

[45] High A. A., Devlin R. C., Dibos A., Polking M., Wild D. S., Perczel J., de Leon N. P., Lukin M. D., Park H. (2015). Visible-Frequency
Hyperbolic Metasurface. Nature.

[46] Raab C., Rieger J., Ghosh A., Spellberg J. L., King S. B. (2024). Surface Plasmons in Two-Dimensional MXenes. J. Phys. Chem. Lett..

[47] Hu T., Zhang H., Wang J., Li Z., Hu M., Tan J., Hou P., Li F., Wang X. (2015). Anisotropic Electronic
Conduction in Stacked Two-Dimensional Titanium Carbide. Sci. Rep..

[48] Garbrecht M., Hultman L., Fawey M. H., Sands T. D., Saha B. (2018). Tailoring
of Surface Plasmon Resonances in TiN/(Al_0.72_Sc_0.28_)N Multilayers by Dielectric Layer Thickness Variation. J. Mater. Sci..

[49] Riis-Jensen A. C., Lu J., Thygesen K. S. (2020). Electrically
Controlled Dielectric Band Gap Engineering
in a Two-Dimensional Semiconductor. Phys. Rev.
B.

[50] Alonso M. I., Marcus I. C., Garriga M., Goñi A. R., Jedrzejewski J., Balberg I. (2010). Evidence of Quantum Confinement Effects
on Interband Optical Transitions in Si Nanocrystals. Phys. Rev. B.

[51] DeCrescent R. A., Venkatesan N. R., Dahlman C. J., Kennard R. M., Chabinyc M. L., Schuller J. A. (2019). Optical Constants and Effective-Medium
Origins of Large
Optical Anisotropies in Layered Hybrid Organic/Inorganic Perovskites. ACS Nano.

