# Supplementary material for: Tuning Anisotropic Optical Properties of Inorganic and Hybrid Organic–Inorganic MXenes via Topochemical Surface Modification
Source: Nano Lett. 2026 Jul 22;26(32):10919–26. doi: 10.1021/acs.nanolett.6c03430 (PMC13495941; doi:10.1021/acs.nanolett.6c03430)
Supplement: Supplementary file 1 [file nl6c03430_si_001.pdf]

Supporting Information

Tuning Anisotropic Optical Properties of Inorganic and Hybrid Organic–Inorganic MXenes via Topochemical Surface Modification

Young-Hwan Kim[1#], Hui Fang[2#*], Hanaul Noh[3], Abu Rashed Md. Shawon[4,5], Sanguk Han[6], Daniel Intriago[6], Teng Zhang[7], Ashley E. Cavanagh[8], Gusaimas M. H. H. Akbar[9], Francisco Lagunas[8,9], Yury Gogotsi[7*], Aleksandra Vojvodic[6*], Aaron J. Rossini[4,5*], Zahra Fakhraai[2*], Dmitri V. Talapin[1,10,11*]

[1] *Pritzker School of Molecular Engineering, University of Chicago, Chicago, IL 60637, USA*

[2] *Department of Chemistry, University of Pennsylvania, Philadelphia, PA 19104, USA*

[3] *Park Systems Inc., Santa Clara, CA 95054, USA*

[4] *Department of Chemistry, Iowa State University, IA 50011, USA*

[5] *Division of Materials Science and Engineering, Ames National Laboratory, IA 50011, USA*

[6] *Department of Chemical and Biomolecular Engineering, University of Pennsylvania, PA 19104, USA*

[7] *Department of Materials Science and Engineering and A.J. Drexel Nanomaterials Institute, Drexel University, Philadelphia, PA 19104, USA*

[8] *Department of Mechanical Engineering and Materials Science, Washington University in St. Louis, St. Louis, MO 63130, USA*

[9] *Institute for Material Science and Engineering, Washington University in St. Louis, St. Louis, MO 63130, USA*

[10] *Department of Chemistry and James Franck Institute, University of Chicago, Chicago, IL 60637, USA*

[11] *Center for Nanoscale Materials, Argonne National Laboratory, Lemont, IL 60439, USA*

[#] These authors contributed equally.

[*] Corresponding authors: huifang1@sas.upenn.edu, gogotsi@drexel.edu, alevoj@seas.upenn.edu, arossini@iastate.edu, fakhraai@sas.upenn.edu, dvtalapin@uchicago.edu

This PDF file includes:

Materials and Methods

Supporting Figures S1–S23

Supporting Tables S1–S9

Supporting References

Materials and Methods

Synthesis of Ti_3AlC_2 MAX phase

The synthesis of Ti_3AlC_2 MAX phase precursor was performed using the modified method reported previously.[1] In short, the powders of TiC, Ti, and Al (2:1:1 mass ratio) were ball-milled for 18 h using zirconia balls. The mixture was loaded into an alumina crucible (100 mm × 40 mm × 20 mm) and annealed at 1400 °C for 48 hours. The sintered MAX phase block was immersed in a 9M HCl solution for 48 hours to dissolve the intermetallic and excess Al. The powder was washed to neutral pH and dried under vacuum at 70 °C for 24 hours. The dried MAX phase powder was sieved, and particles with sizes of 38 to 75 μm were collected.

Synthesis of $Ti_3C_2Cl_2$ MXene

$Ti_3C_2Cl_2$ MXene was synthesized using a modified literature procedure.[2] Ti_3AlC_2 MAX phase (0.1–3.0 g) was mixed with $CdCl_2$ salts in a 1:8 molar ratio and transferred to an alumina or silicon nitride crucible. The mixture was heated under Ar at 610 °C for 10 h. The reaction product was treated with concentrated aqueous HCl (37%) to dissolve excess $CdCl_2$ and metallic Cd, then washed repeatedly with deionized water until a neutral pH was achieved. The resulting $Ti_3C_2Cl_2$ MXene powder was dried under vacuum at 40 °C for >12 h prior to further use.

Synthesis of hybrid organic-inorganic MXene (*h*-MXenes)

h-MXenes were synthesized using a modified literature procedure.[3] All reactions were performed under an inert atmosphere in either an N_2-filled glovebox with oxygen and moisture levels below 0.1 ppm. 40 mg of $Ti_3C_2Cl_2$ MXene powder was combined with 1 mL of desired primary amines, and 4-fold molar excess of *n*-BuLi relative to $Ti_3C_2Cl_2$ MXenes. The reaction mixture was heated at an elevated temperature under reflux for a specific duration. After the reaction, the product was washed sequentially with toluene and methanol, then dried under vacuum prior to further use.

X-ray diffraction (XRD)

Powder X-ray diffraction patterns in reflection mode were collected using a Rigaku benchtop diffractometer equipped with a HyPix-400 MF two-dimensional hybrid pixel array detector (HPAD) and a Cu Kα radiation source (λ = 1.5418 Å), operated at 40 kV and 15 mA.

Scanning electron microscopy-energy dispersive X-ray spectroscopy (SEM-EDS)

SEM imaging and EDS elemental mapping were performed using a Carl Zeiss Merlin field-emission scanning electron microscope (FE-SEM) equipped with Oxford Ultim Max 100 silicon drift detectors (SDD). The accelerating voltage was set to 10 kV with a beam current of 1 nA. For halogen quantification by SEM–EDS, measurements from at least three different spots and magnifications were averaged.

Scanning transmission electron microscopy (STEM)

Ti_3AlC_2 and $Ti_3C_2Cl_2$ samples were prepared for cross-sectional STEM imaging using a ThermoFisher Scios 2 DualBeam focused ion beam (FIB). Individual flakes of each sample were coated in a 2 µm protective platinum layer and then lamellae were cut from each with a 30 keV gallium ion beam. The lamellae were lifted out with a ThermoFisher Easylift Nanomanipulator and each attached to a copper half-grid with a platinum patch. The lamellae were then thinned until they became electron-transparent. Initial thinning was done with a 30 kV ion beam, then reduced to 16 kV, 8 kV, 5 kV, and finally 2 kV for final polishing. STEM imaging of Ti_3AlC_2 and $Ti_3C_2Cl_2$ was performed using a JEOL Grand ARM 2 aberration-corrected STEM, operated at 200 kV. High-angle annular dark field (HAADF) images ofTi_3AlC_2 were taken with collection angles ranging from 54–220 mrad. Low-angle annular dark field (LAADF) images of $Ti_3C_2Cl_2$ were acquired with collection angles ranging from 27–110 mrad.

The $Ti_3C_2(dda)_{2/3}$ *h*-MXene was imaged with a JEOL ARM 200CF aberration-corrected STEM that was operated at 200 kV. LAADF images were taken with collection angles ranging from 30–120 mrad. The sample was prepared for STEM imaging by drop-casting $Ti_3C_2(dda)_{2/3}$ flakes onto a copper grid.

Imaging spectroscopic ellipsometry measurements

Imaging spectroscopic ellipsometry was performed using a Park Systems Accurion EP4 imaging ellipsometer over 300–1700 nm spectral range at a fixed incidence angle of 45°. Drop-cast particles on a Si wafer were first located and oriented using the integrated optical microscope: specimens lying flat on the substrate were selected for the (0°, 0°, 0°) configuration, whereas particles standing on edge were used for the (90°, 90°, 0°) and (0°, 90°, 0°) configurations, as shown in Figure 2 in the main text. For the latter two, measurements on the same crystallite were acquired by rotating the substrate and re-aligning the beam to the same spot. To ensure robustness, each configuration was measured on at least three distinct particles, and the measurement was targeted to a flat, highly reflective region of each crystallite.

Spectroscopic ellipsometry data analysis

The spectroscopic ellipsometry (SE) data measured with an Accurion EP4 ellipsometer were first converted to a format compatible with the CompleteEase software (J. A. Woollam, version 6.75b-IR-NMSU), which was used for data analysis. The measured and fitted SE angles Ψ(λ), Δ(λ) of $Ti_3C_2Cl_2$ and *h*-MXenes, acquired under both (90°, 90°, 0°) and (0°, 90°, 0°) configurations at room temperature, are displayed in Figure S6. During the fitting process, the MXenes were treated as substrate layers.

To enhance fitting accuracy, a multi-sample analysis method was employed. Specifically, the SE data from the (90°, 90°, 0°) and (0°, 90°, 0°) configurations were fitted globally using a uniaxial anisotropic model. A Kramers-Kronig constrained B-spline model was used to estimate the in-plane and out-of-plane dielectric dispersion of the samples. Then, a generalized oscillator (Gen-Osc) model combining

1) Three harmonic oscillators and one Drude oscillator for in-plane $Ti_3C_2Cl_2$
2) Two harmonic oscillators and one Drude oscillator for out-of-plane $Ti_3C_2Cl_2$
3) Five harmonic oscillators for in-plane *h*-MXenes
4) Two harmonic oscillators for out-of-plane *h*-MXenes

was used to parameterize the imaginary part (ε_2) of the dielectric function obtained by an initial point-by-point B-Spline fitting. The functions are given as

$$\varepsilon_{Harmonic}(E) = \frac{A_n Br_n}{2}\left(\frac{1}{E_n - E - i\frac{1}{2}Br_n} + \frac{1}{E_n + E - i\frac{1}{2}Br_n}\right) \quad \text{(S1)}$$

$$\varepsilon_{Drude(RT)}(E) = \frac{-\hbar^2}{\varepsilon_0 \rho_n (\tau_n \cdot E^2 + i\hbar E)} \quad \text{(S2)}$$

where E (eV) is the energy, which can be related to the wavelength λ (nm) by $E=hc/\lambda$. A_n is the unitless amplitude, and Br_n, and E_n are the full width at half-maximum (FWHM or breadth, eV), and the center energy (eV), respectively. ε_0, ρ_n, and τ_n are the permittivity of the free space (vacuum dielectric constant) (F×cm^{-1}), the resistivity (W×cm), and the mean scattering time (fs), respectively.[30]

The real part (ε_1) of the dielectric constant was then fitted by fitting the value of the dielectric constant at the high energy limit (E_{inf} or $\varepsilon_{¥}$), potential resonances in the UV region (UV pole), and potential resonances in the IR region (IR pole). $\varepsilon_{¥}$ represents the ε_1 value "at infinity", which accounts for the effects of absorption far outside the measured spectral range. Poles are equivalent to Lorentz oscillators with zero broadening. The UV and IR poles describe absorptions that occur at higher and lower energies outside the measured spectral range, respectively. As such, they only affect ε_1.[30]

$$\varepsilon_{UV\_pole}(E) = \frac{A_{n_{UV}}}{E_{n_{UV}}{}^{2} - E_{UV}^{2}} \quad \text{(S3)}$$

$$\varepsilon_{IR\_pole}(E) = \frac{-A_{n_{IR}}}{E_{IR}^{2}} \quad \text{(S4)}$$

where ε_{UV_pole} is ε_2 of the UV pole as a function of E, E_{nUV} (eV) and A_{nUV} (eV^2), are the center energy and amplitude of the UV pole; ε_{IR_pole} is ε_2 of the IR pole as a function of E, E_{IR} (= 10^{-8} eV) and A_{nIR} (eV^2), are the energy and the amplitude of the IR pole.

The new model was then fitted to the original data to improve fitting accuracy. The resistivity and scattering time of samples can be deduced from the Drude oscillator. The fitting results are tabulated in Tables S1, S2, S3, S6, S8, and S9. The deduced ε_1 and ε_2 spectra are plotted in Figures 4b–e, 5, S7, and S8. The corresponding real part (*n*) and imaginary part (*k*, or extinction coefficient) for the index of refraction are plotted in Figures S9, S10, and S16.

To further test the reliability of our models, the same model was used to fit the individual (90°, 90°, 0°), (0°, 90°, 0°), and (0°, 0°, 0°) configuration data. The results shown in Figures S21–23 were comparable with the results globally fitting both (90°, 90°, 0°) and (0°, 90°, 0°) configurations. (Note that an isotropic model, only including the in-plane dielectric constants, was enough to fit the (0°, 0°, 0°) data.) More detailed fitting procedures for similar $Ti_3C_2T_x$ and $Ti_3C_2Cl_2$ MXenes can be found in our previous work,[4–7] where only the in-plane dielectric constant was measured and fitted.

Alternative fitting models for *h*-MXenes are also tested, and the results are shown in Figures S15 and S18. For example, 4 harmonic and 1 Drude oscillators were used to fit the in-plane ε_2 of $Ti_3C_2(bua)_{2/3}$ MXene to deduce the resistivity (Figure S15). The fitting results are tabulated in Table S4. A transparent Cauchy dispersion equation (equation S5) was used to fit the out-of-plane e_1 of both $Ti_3C_2(bua)_{2/3}$ and $Ti_3C_2(oca)_{2/3}$ MXene, where the ε_2 is assumed to be zero (S18).[16] The fitting results are tabulated in Tables S5 and S7.

$$n(\lambda) = A + \frac{B}{\lambda^2} + \frac{C}{\lambda^4} \quad \text{(S5)}$$

where *n* is the index of refraction, λ is the wavelength (mm), A, B, and C are fitting parameters.

<u>Solid-state nuclear magnetic resonance (ssNMR) measurements</u>

^{1}H and ^{15}N magic angle spinning (MAS) solid-state NMR experiments were performed on a Bruker wide-bore 14.1 T [ν_0(^{1}H) = 600 MHz] NMR spectrometer equipped with a Bruker AVANCE NEO console. The butylamine terminated MXene sample was packed into 1.3 mm zirconia rotors in a dry nitrogen glovebox. All experiments were performed under 50 kHz MAS frequency with a 1.3 mm HXY triple resonance

Phoenix MAS probe configured in ^{1}H-^{13}C-^{15}N mode. The ^{1}H and ^{15}N radiofrequency (RF) fields were directly calibrated on the sample. ^{1}H RF fields were calibrated by observing nutation profiles using a pulse-acquire sequence and varying the duration of the excitation pulse. The ^{1}H RF field was then confirmed by using a spin-lock pulse sequence to observe rotary resonance conditions ($\nu_1 = 0.5 \times \nu_{rot}$). ^{15}N RF fields were approximately estimated by varying the $\pi/2$ pulse durations within the ^{1}H-detected ^{15}N CP-HETCOR NMR experiment. $^{1}H \rightarrow ^{15}N$ CP conditions were directly optimized on the sample. A ^{1}H saturation recovery NMR experiment was used to measure the ^{1}H longitudinal relaxation time constant (T_1). ^{1}H T_1 of 1.92 s was measured for butylamine-terminated MXene; consequently, recycle delays of 2.5 s were used for all subsequent NMR experiments on the 14.1 T magnet. The recycle delay corresponds to approximately $1.3 \times T_1$ and was used to maximize sensitivity.[8]

The ^{1}H spin echo SSNMR spectrum of butylamine terminated MXene was acquired with 100 kHz ^{1}H RF pulses, a rotor-synchronized echo delay, 16 scans, and a recycle delay of 2.5 s. 2D $^{1}H\{^{15}N\}$ CP-HETCOR[9,10] NMR experiments were performed with a forward CP contact time of 3 ms, and 2D NMR spectra were acquired with a back CP contact time of 3 ms or a shorter back CP contact time of 0.5 ms to probe directly bonded NH pairs. The STATES-TPPI method was used to achieve frequency discrimination and obtain absorptive peaks.[11] 160 scans were acquired for each t_1-increment. The t_1-evolution time was incremented in steps of 20 µs and 64 t_1-increments were acquired. During t_1-evolution period, continuous wave ^{1}H heteronuclear coupling was performed with a 25 kHz RF field. The $^{1}H \rightarrow ^{15}N$ CP transfers on the sample used ^{1}H and ^{15}N spin-lock RF fields of 85 kHz and 30 kHz. During CP spin-lock experiments, a 90% to 100% linear amplitude ramp was applied on the ^{1}H channel spin-lock pulse to broaden the Hartmann-Hahn match condition.[12]

Direct excitation ^{13}C Carr-Purcell Meiboom-Gill (CPMG)[13] solid-state NMR experiments were performed on a Bruker wide-bore 9.4 T [$\nu_0(^{1}H)$ = 400 MHz] NMR spectrometer equipped with a Bruker AVANCE III HD console. The CPMG pulse sequence was synchronized to the sample rotation. Bruker 1.3 mm resonance probe in HX double mode was used for the ^{13}C CPMG NMR experiments. The direct excitation ^{13}C CPMG SSNMR spectra of both samples (butylamine-terminated MXene and $Ti_3C_2Cl_2$) were acquired with a 50 kHz MAS frequency. For butylamine terminated MXene, a recycle delay of 5 s, 20 spin echoes were acquired with an echo duration of 0.8 ms, ^{13}C π-refocusing pulses were 4 µs in duration, corresponding to a RF power of 125 kHz, and 16384 scans were used. ^{1}H heteronuclear decoupling was achieved with 25 kHz continuous wave (CW) irradiation. For $Ti_3C_2Cl_2$, a recycle delay of 3 s, 20 spin echoes were acquired with an echo duration of 2 ms, ^{13}C π-refocusing pulses were 6 µs in duration corresponding to a RF power of 83 kHz and 16384 scans were used.

All NMR spectra were processed in TopSpin 3.8.0 software. ^{1}H chemical shifts were referenced to neat tetramethylsilane using adamantane (δ_{iso} (^{1}H) = 1.82 ppm) as a secondary chemical shift reference. Previously published relative NMR frequencies were used to indirectly reference the ^{13}C and ^{15}N NMR spectra.[14]

Density functional theory (DFT) calculations

DFT calculations were performed using the Quantum Espresso (QE) plane wave code.[15–17] BEEF-vdW functional[18] and SG15 Optimized Norm-Conserving Vanderbilt (ONCV) pseudopotentials[19,20] were used for the calculation. The ground state charge density was first converged from the self-consistent field (SCF) calculations, followed by non-self-consistent field (NSCF) calculations with denser *k*-point sampling. The wavefunctions resulting from the NSCF calculations were then used to calculate the band structure or the dielectric function using *bands.x* or *epsilon.x* module. To reflect the experimental coverage of each termination, butylamido and butylimido-terminated *h*-MXenes were modeled in 3×3×1 supercells with periodic boundary conditions applied in the *x*, *y*, *z* directions to properly capture the experimental coverage of alkylamido and alkylimido ligands.[3]

The band structure was calculated along the high 2D symmetry path ΓKMΓ for each MXene monolayers with 10Å of vacuum along the z-direction. For the Cl– and H–terminated systems, an equal-weight path of 1200 *k*-points was used to generate the band structure. For the butylamido and butylimido terminated systems, an equal-weight path of 180 *k*-points were used as these *h*-MXene models contain larger supercells. Although the *h*-MXene systems correspond to the smallest periodic cells for the chosen ligand coverage and stoichiometry, their larger real-space periodicity leads to a folded band structure relative to the smaller Cl– and H–terminated systems.

The dielectric function of $Ti_3C_2Cl_2$ was calculated using the multilayer $P6_3/mmc$ $Ti_3C_2Cl_2$. SCF and NSCF calculations employed 9×9×3 and Γ-centered 58×58×8 *k*-point grids, respectively.

Effective medium theory (EMT) calculations of *h*-MXenes

EMT calculations were performed following the previous report.[21] Each *h*-MXene was modeled as a periodic stack of two layers, a Ti_3C_2-based inorganic layer (medium 1) and an alkyl-based organic layer (medium 2). The effective in-plane and out-of-plane permittivities of the layered medium can be computed from the intrinsic layer permittivies as follows:

$$\bar{\varepsilon}_{\parallel} = f_1 \cdot \varepsilon_{1,\parallel} + (1 - f_1) \cdot \varepsilon_{2,\parallel} \qquad \text{(S6)}$$

$$\bar{\varepsilon}_{\perp} = (\frac{f_1}{\varepsilon_{1,\perp}} + \frac{f_2}{\varepsilon_{2,\perp}})^{-1} \quad \text{(S7)}$$

Here, f_1 is the volumetric fill fraction of the inorganic layer, and ε_1, ε_2 are the intrinsic permittivity tensor of the inorganic and organic layers, respectively.

The inorganic fill fraction f_1 of each MXene was obtained from the ratio between the Ti_3C_2 slab thickness and the interlayer spacing. The Ti_3C_2 slab thickness was obtained from the cross-sectional STEM image of $Ti_3C_2(dda)_{2/3}$, and the interlayer spacing for each material was determined from the (0002) reflections in the PXRD patterns. This procedure assumes that the inorganic slab thickness is invariant across the series.

The organic layer (medium 2) was treated as a nonabsorbing dielectric medium with $\varepsilon_2 = 2.25$ ($n = 1.5$) for all EMT calculations, consistent with reported refractive indices of alkyl-based organic layers in EMT calculations.[21] The intrinsic permittivity tensor of the inorganic layer (medium 1; ε_1) was obtained by inverting the EMT relations (S6, S7) using the experimentally fitted dielectric function of $Ti_3C_2(bua)_{2/3}$ as $\bar{\varepsilon}$. $Ti_3C_2(bua)_{2/3}$ was chosen as the reference as it gave the lowest MSE in the spectroscopic ellipsometry fitting among the *h*-MXene series (Tables S3 and S4). Using the fill fraction of $Ti_3C_2(bua)_{2/3}$ and $\varepsilon_2 = 2.25$, ε_1 can be back-calculated. The resulting ε_1 was then used as a fixed inorganic-layer input for EMT calculations of the other *h*-MXenes.

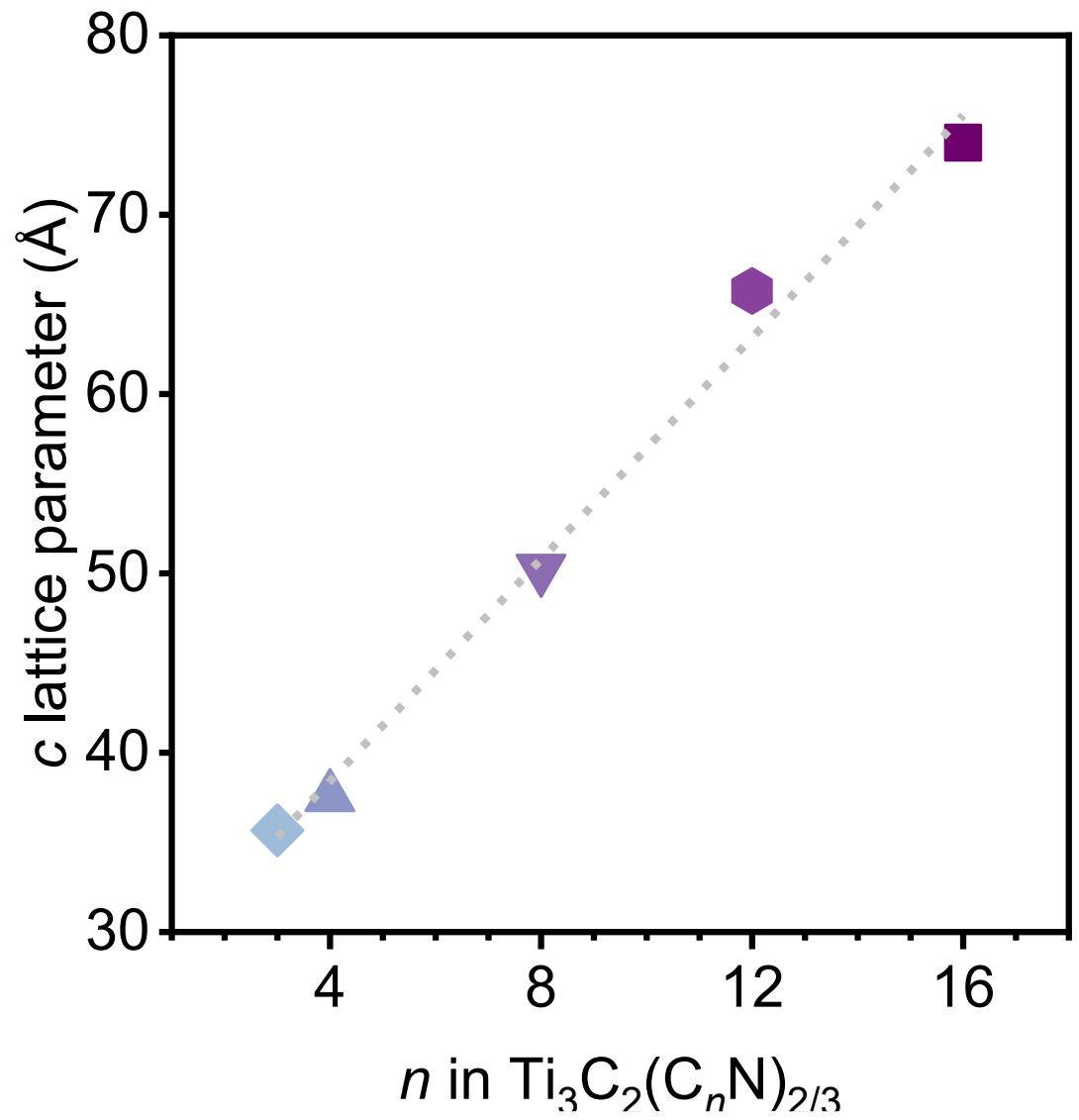

Figure S1. **Changes of the *c* lattice parameter versus number of carbon atoms in the interlayer alkyl groups.** The lattice parameters are determined by the positions of lamellar peaks from PXRD shown in Figure 1i.

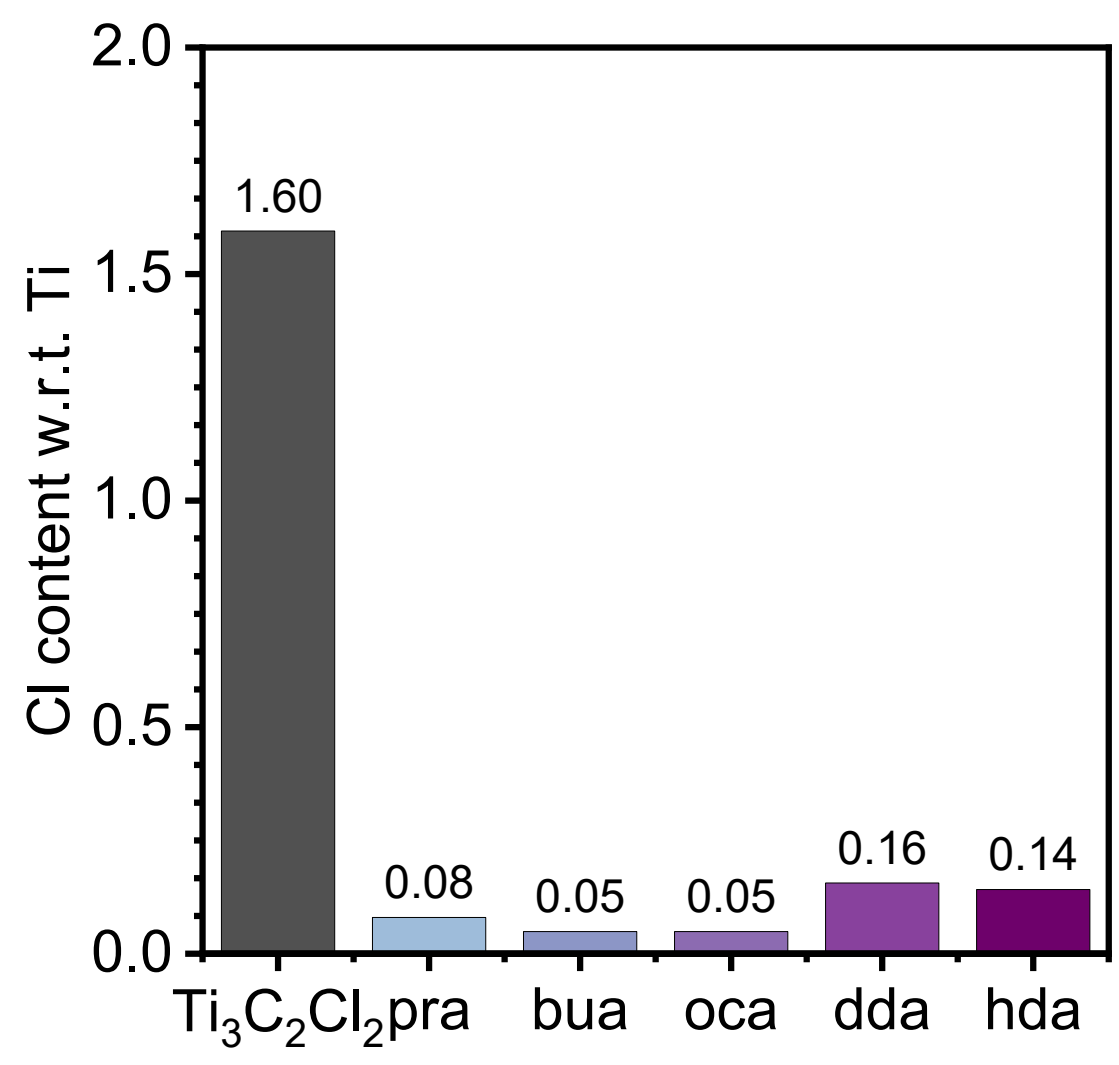

Figure S2. **SEM-EDS analysis of $Ti_3C_2Cl_2$-derived MXenes.** Ensemble Ti:Cl ratios showing substantial loss of surface Cl terminations following the reaction.

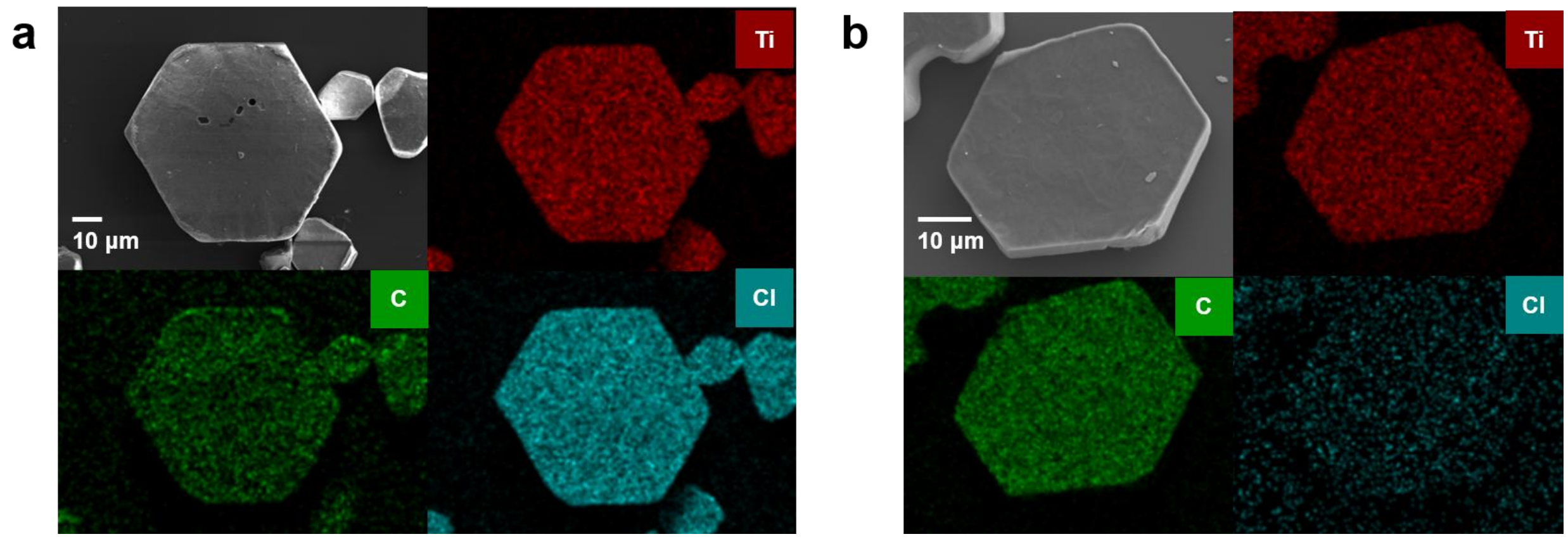

Figure S3. **SEM imaging andEDS mapping of $Ti_3C_2Cl_2$ and $Ti_3C_2(pra)_{2/3}$ MXenes.** Elemental mapping of the (a) $Ti_3C_2Cl_2$ MXene particle shown in Figure 1c and (b) $Ti_3C_2(pra)_{2/3}$ MXenes after substitution. The maps show substantial Cl loss following the reaction.

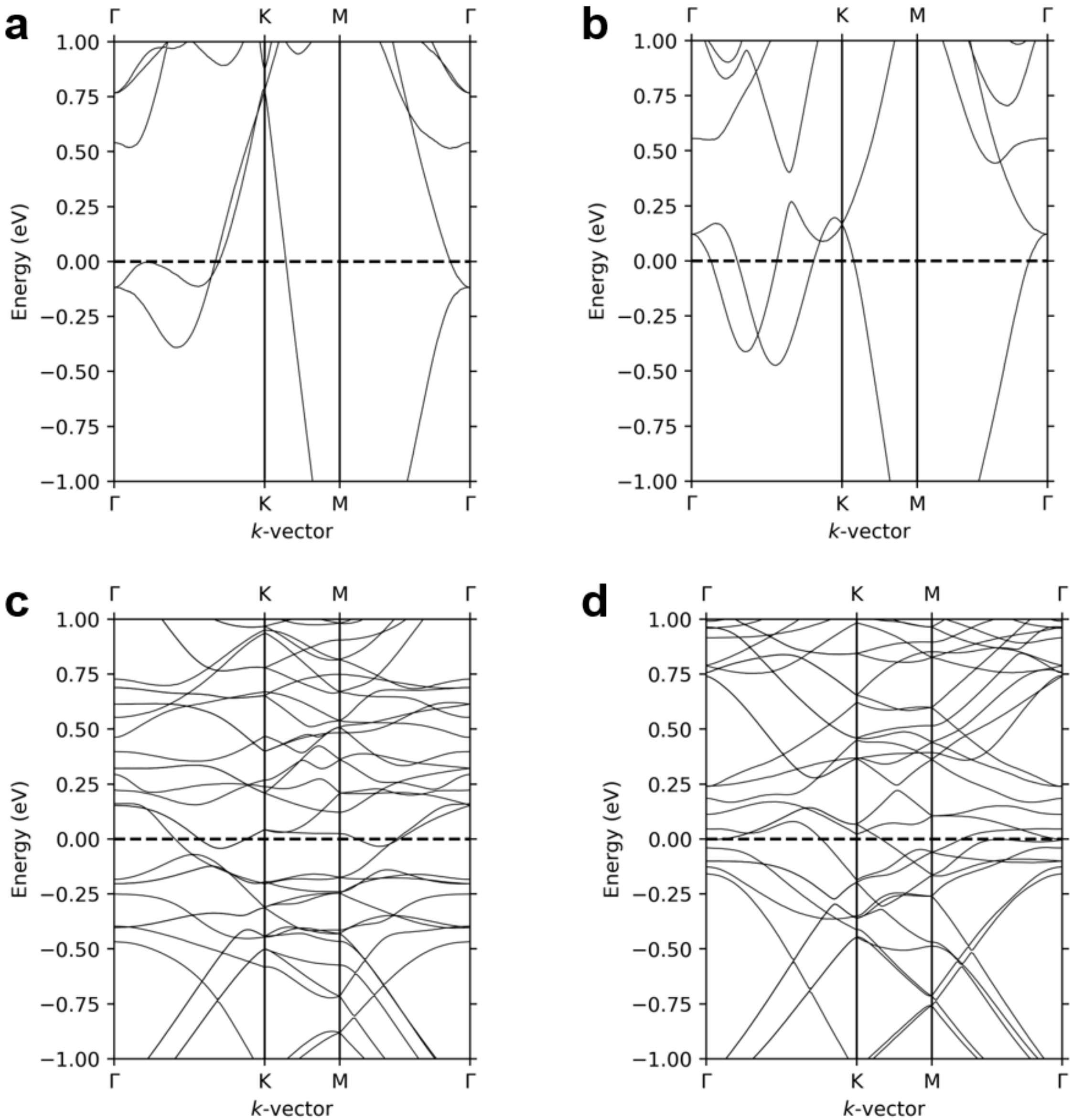

Figure S4. **DFT-calculated electronic band structures.** DFT-calculated band structures of (a) $Ti_3C_2Cl_2$, (b) $Ti_3C_2H_2$, (c) $Ti_3C_2(butylamido)_{2/3}$, and (d) $Ti_3C_2(butylimido)_{2/3}$.

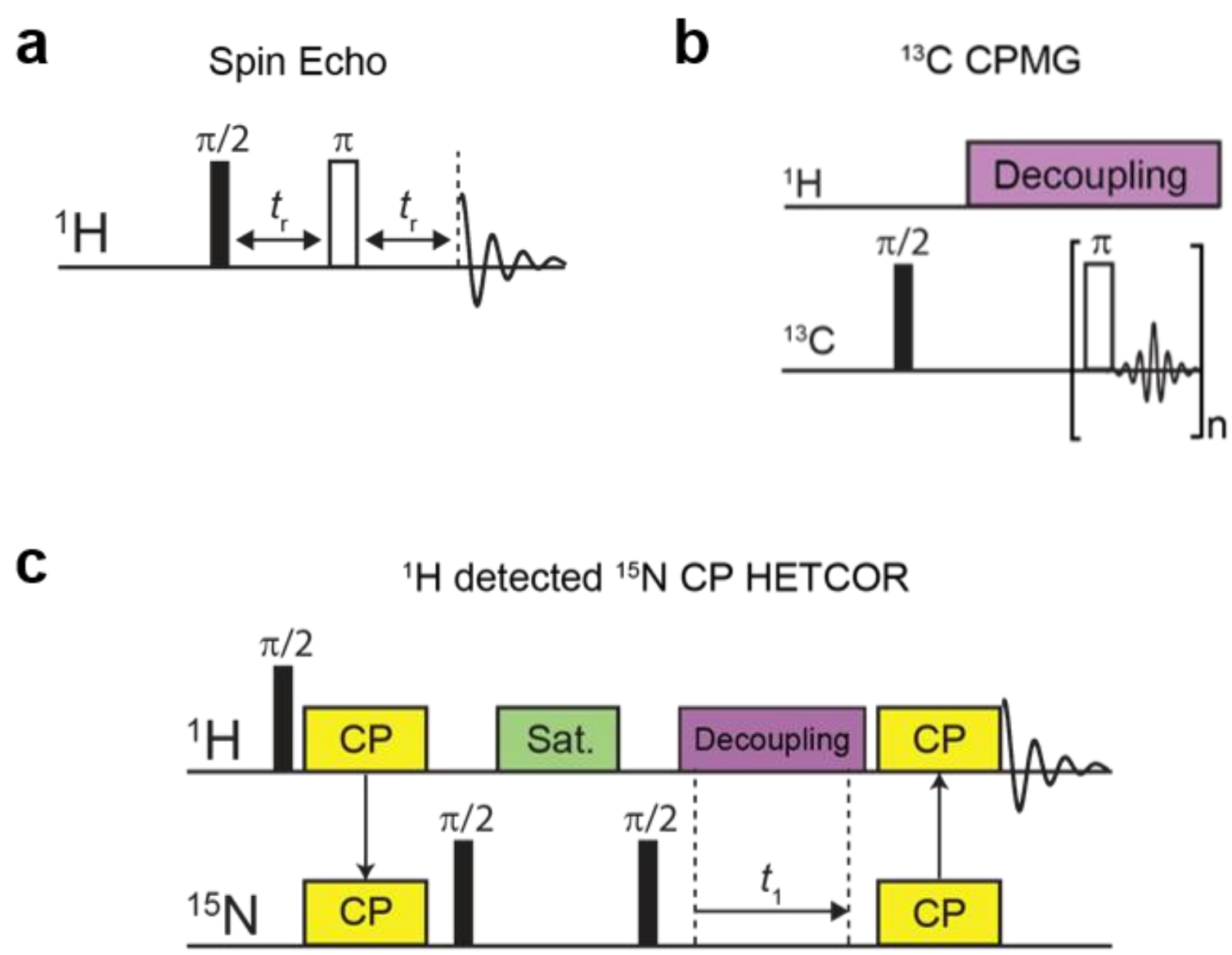

Figure S5. **ssNMR pulse sequences.** Pulse sequences used for the (a) ^{1}H spin echo, (b) ^{13}C CPMG, and (c) ^{1}H-detected ^{15}N CP-HETCOR experiments.

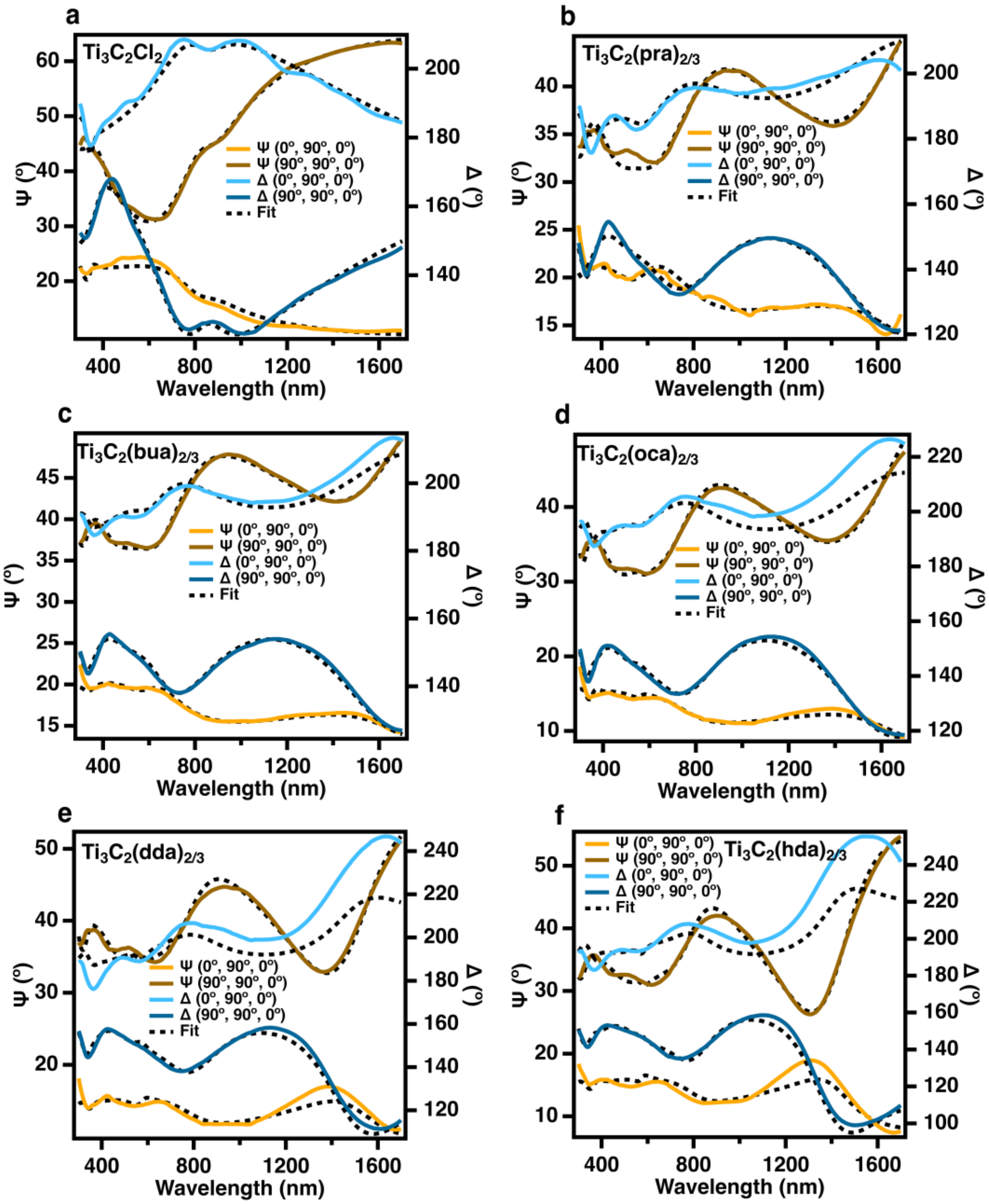

Figure S6. **Global fitted ellipsometry results of $Ti_3C_2Cl_2$ and *h*-MXenes under measurements (90°, 90°, 0°) and (0°, 90°, 0°).** Measured Ψ and Δ spectra, together with the global best fits (dashed line) as a function of wavelength at angle of incident 45° for (a) $Ti_3C_2Cl_2$, (b) $Ti_3C_2(pra)_{2/3}$, (c) $Ti_3C_2(bua)_{2/3}$, (d) $Ti_3C_2(oca)_{2/3}$, (e) $Ti_3C_2(dda)_{2/3}$, and (f) $Ti_3C_2(hda)_{2/3}$. The fitting results are tabulated in Tables S1, S2, S3, S6, S8, and S9. A global fitting approach was performed by simultaneously fitting the data collected in the (90°, 90°, 0°) and (0°, 90°, 0°) configurations. An anisotropic uniaxial model was used for fitting.

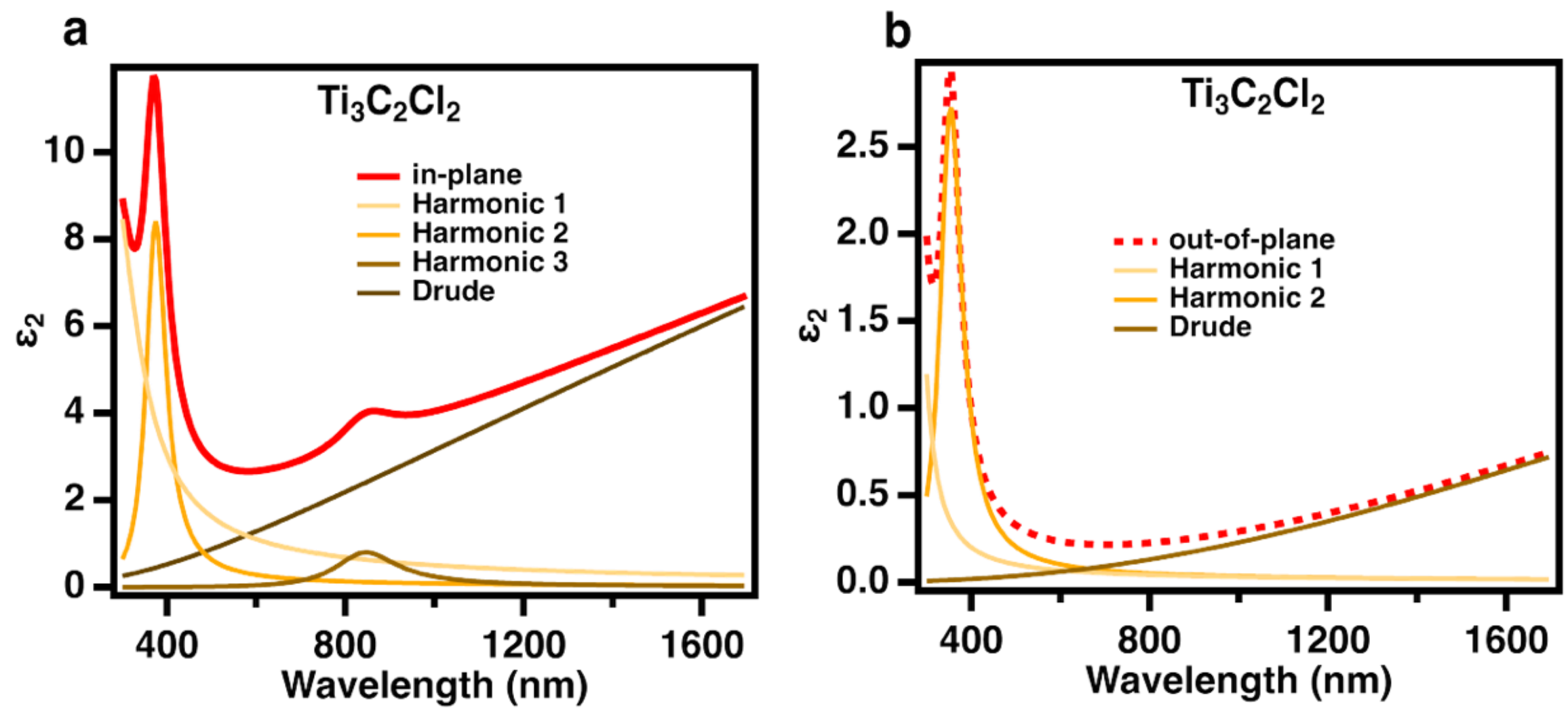

Figure S7. **Oscillators to construct the imaginary parts of the dielectric constant of $Ti_3C_2Cl_2$ MXene.** The (a) in-plane and (b) out-of-plane real and imaginary parts of the dielectric constants (e_2) as a function of wavelength for $Ti_3C_2Cl_2$ MXene. The orange curves represent the separate contributions of the oscillators used to construct ε_2.

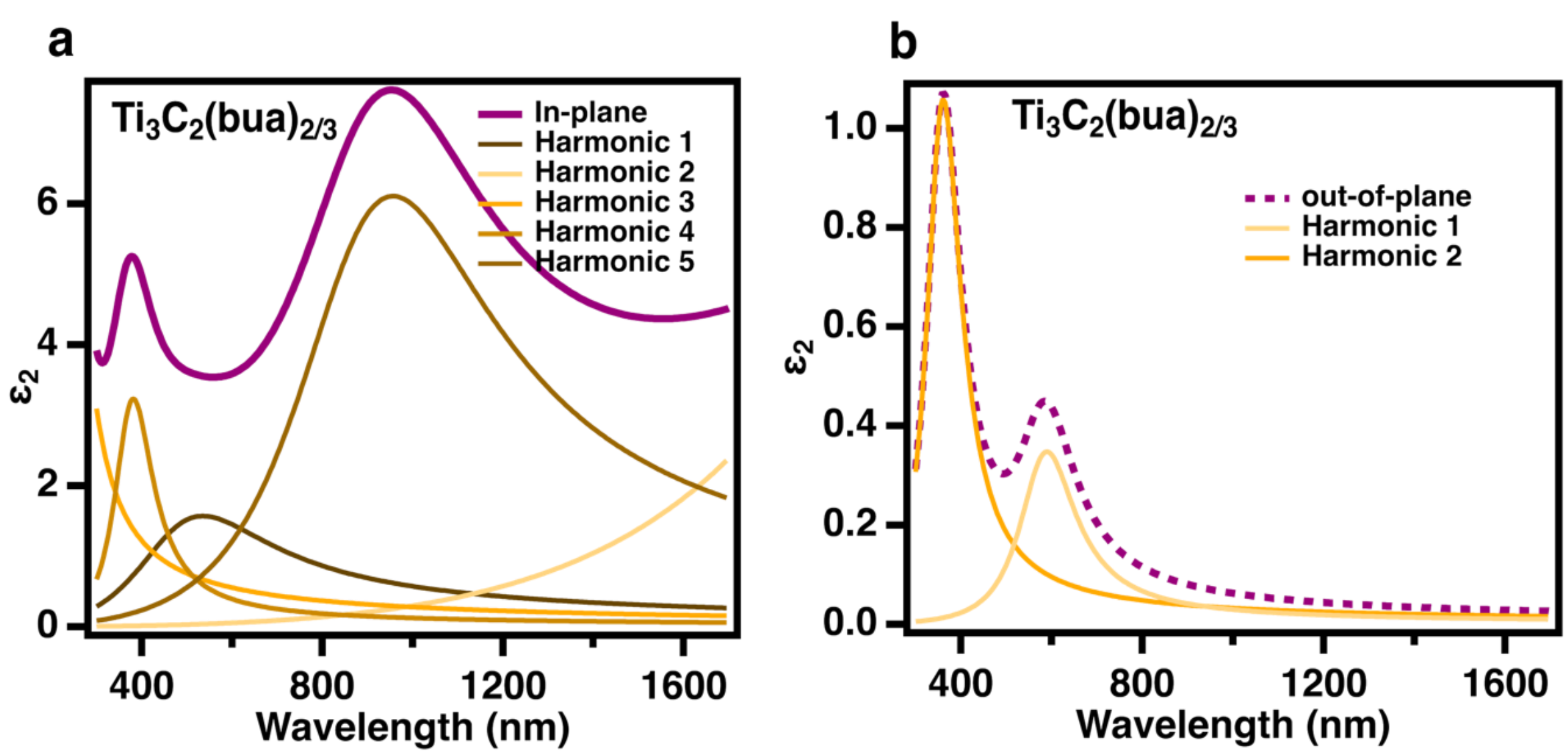

Figure S8. **Oscillators to construct the imaginary parts of the dielectric constant of $Ti_3C_2(bua)_{2/3}$ MXene.** The (a) in-plane and (b) out-of-plane real and imaginary parts of the dielectric constants (ε_2) as a function of wavelength for $Ti_3C_2(bua)_{2/3}$ MXene. The orange curves represent the separate contributions of the oscillators used to construct ε_2.

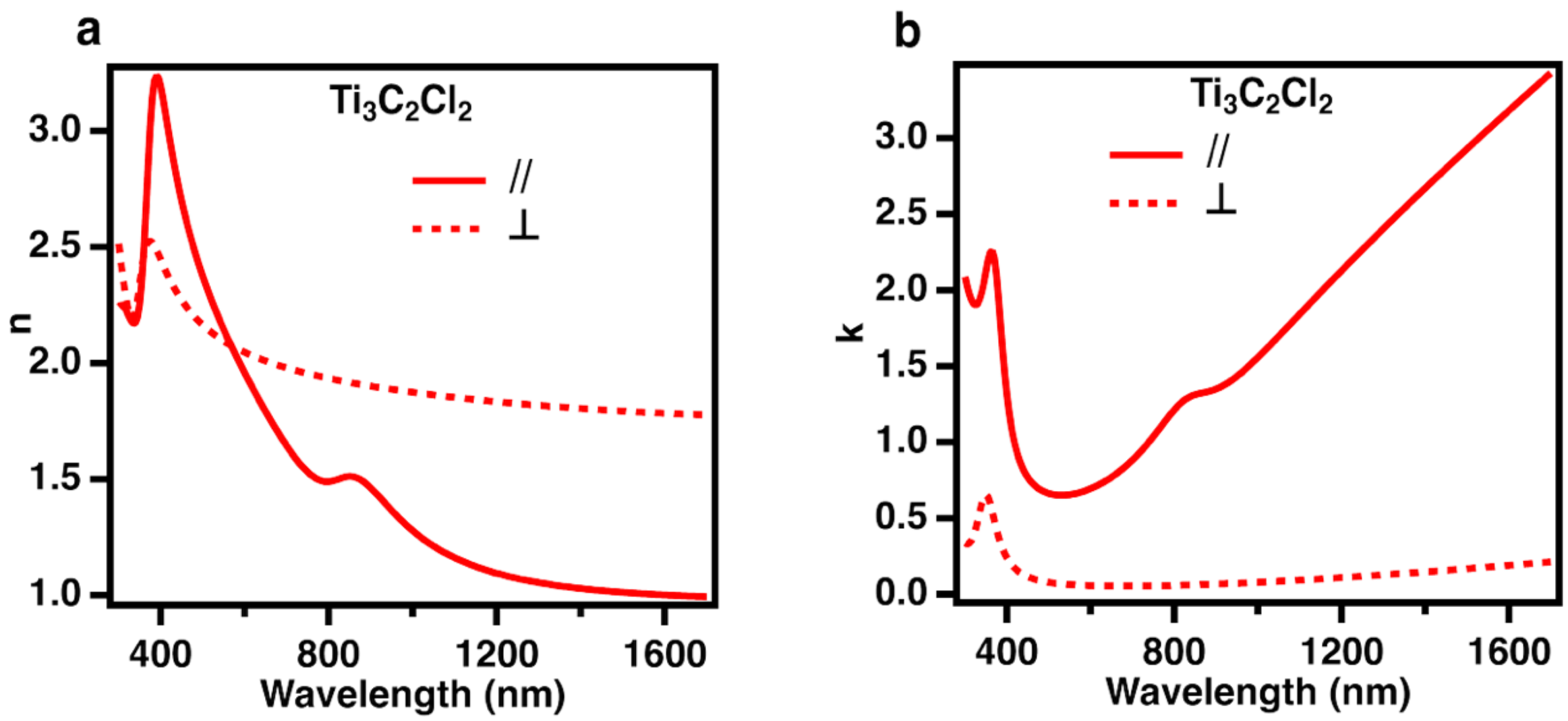

Figure S9. **Refractive index of $Ti_3C_2Cl_2$ MXene.** The (a) real (*n*) and (b) imaginary (*k*) parts of the refractive index as a function of wavelength for $Ti_3C_2Cl_2$ MXene. // and ⊥ denote the in-plane and out-of-plane refractive index.

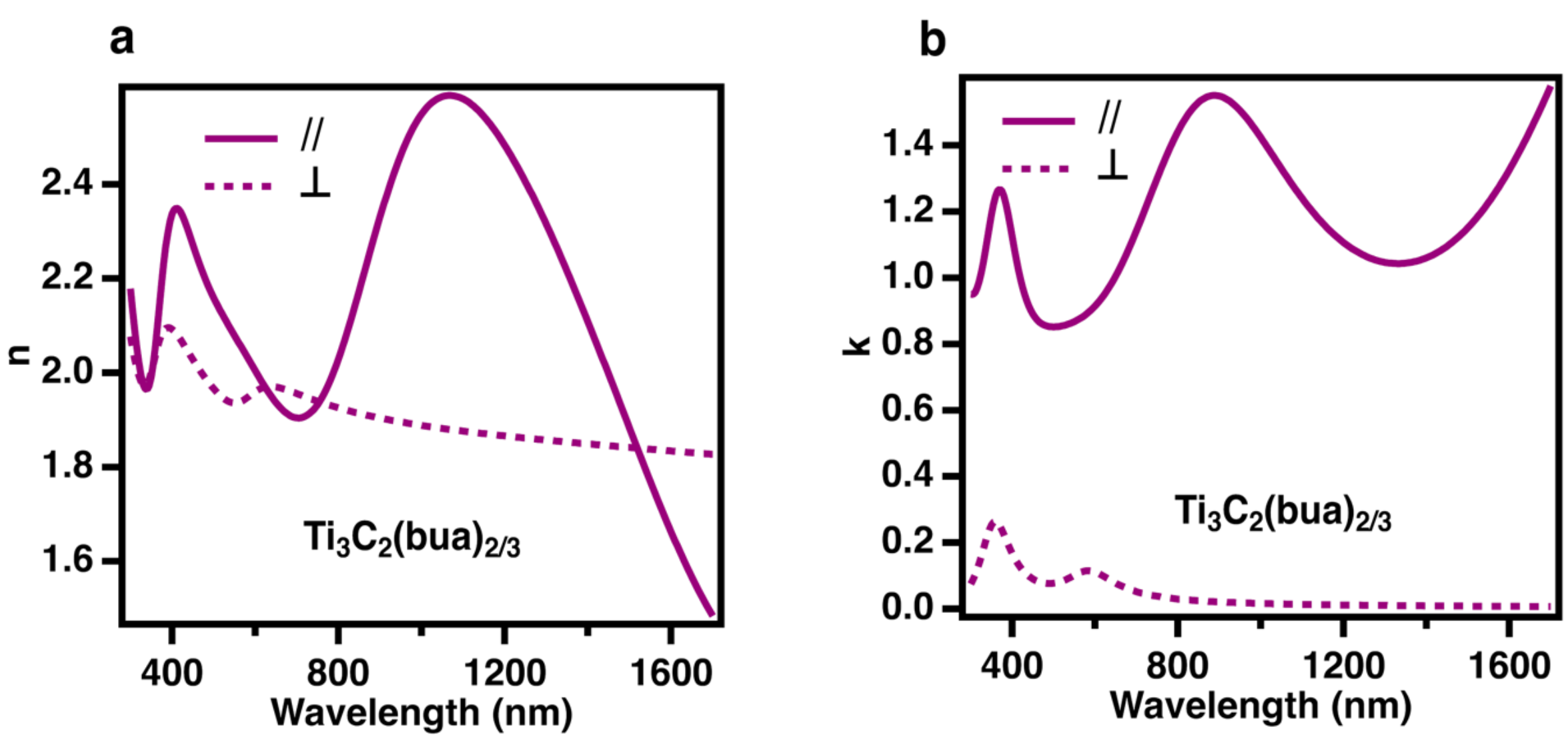

Figure S10. **Refractive index of $Ti_3C_2(bua)_{2/3}$ MXene.** The (a) real (*n*) and (b) imaginary (*k*) parts of the refractive index as a function of wavelength for $Ti_3C_2(bua)_{2/3}$ MXene. // and ⊥ denote the in-plane and out-of-plane refractive index.

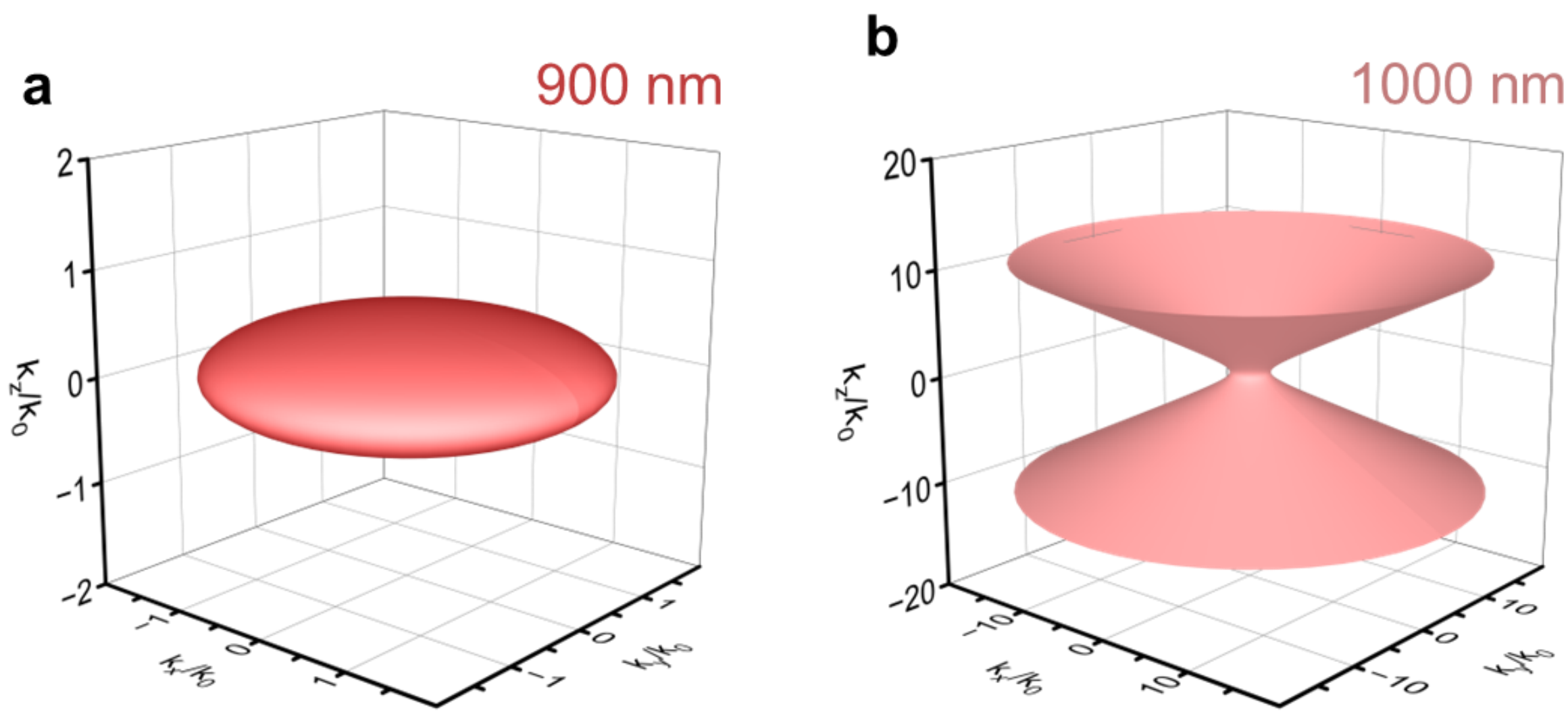

Figure S11. **Topological transition of the isofrequency contours of $Ti_3C_2Cl_2$ MXene.** Isofrequency contours of $Ti_3C_2Cl_2$ MXene calculated at (a) 900 nm and (b) 1000 nm. The isofrequency contours above for transverse magnetic (TM) modes can be calculated by[22]:

$$\frac{{k_x}^2 + {k_y}^2}{\varepsilon_\perp} + \frac{{k_z}^2}{\varepsilon_\parallel} = \frac{w^2}{c^2}$$

where k_x, k_y and k_z are the *x*, *y* and *z* components of the wavevector, ω is the frequency, *c* is the speed of light, and $\varepsilon_{//}$ and $\varepsilon_\perp$ are the measured real parts of the dielectric function (ε_1) of $Ti_3C_2Cl_2$.

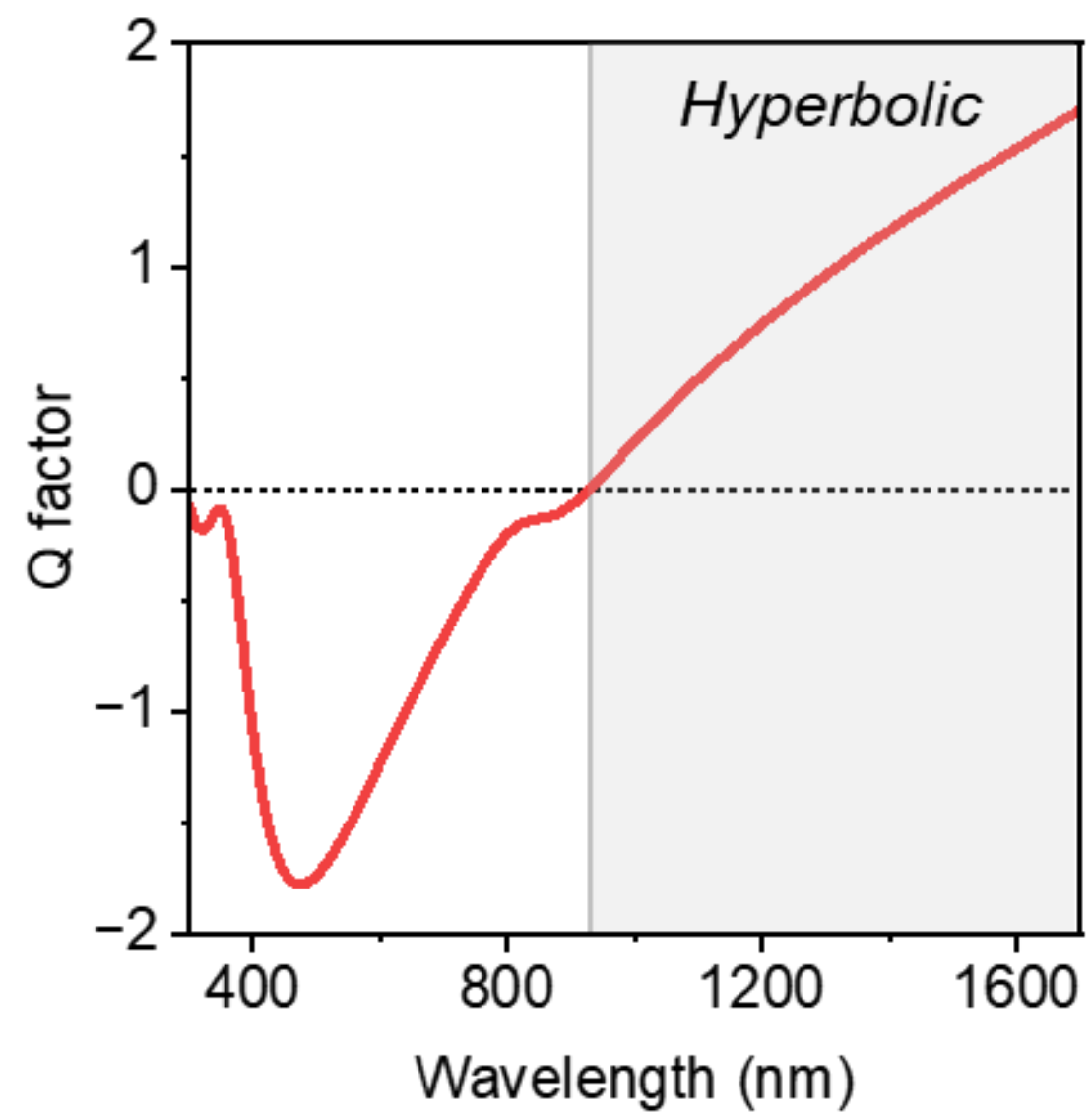

Figure S12. **Hyperbolic quality factor of $Ti_3C_2Cl_2$ MXenes.** Hyperbolic quality factor of $Ti_3C_2Cl_2$ MXenes, calculated using $|\varepsilon_{//}/\varepsilon_{\perp}|$ of ε_1.

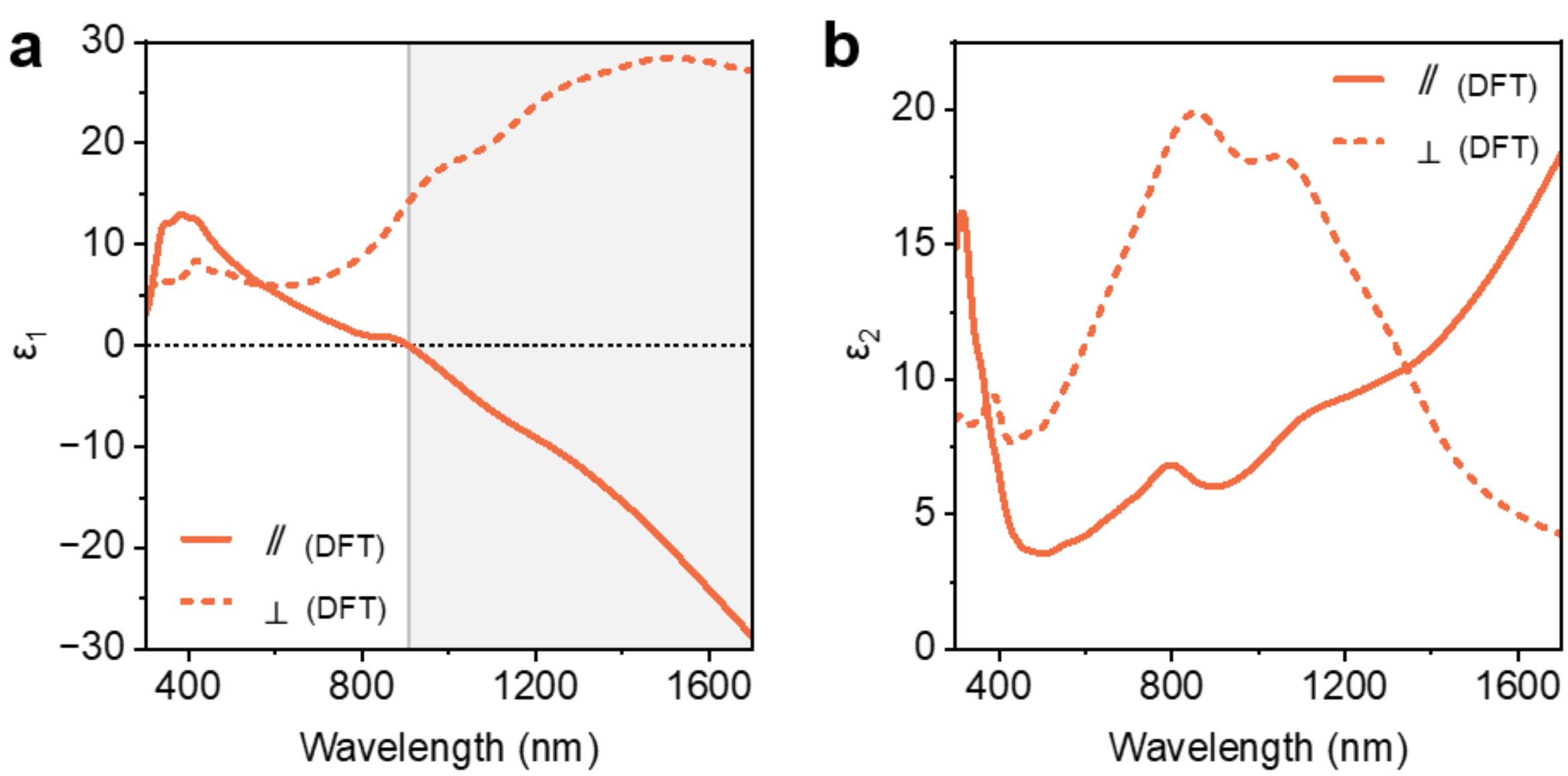

Figure S13. **DFT-calculated permittivity tensor of $Ti_3C_2Cl_2$ MXenes.** (a) Real (ε_1) and (b) imaginary (ε_2) parts of the DFT-calculated dielectric constant as a function of wavelength for $Ti_3C_2Cl_2$. The deviation of the out-of-plane component from experiments likely reflects the previously reported sensitivity of $\varepsilon_{\perp}$ to crystal local-field effects,[23–29] which are not included in the present calculation and warrant further theoretical investigation.

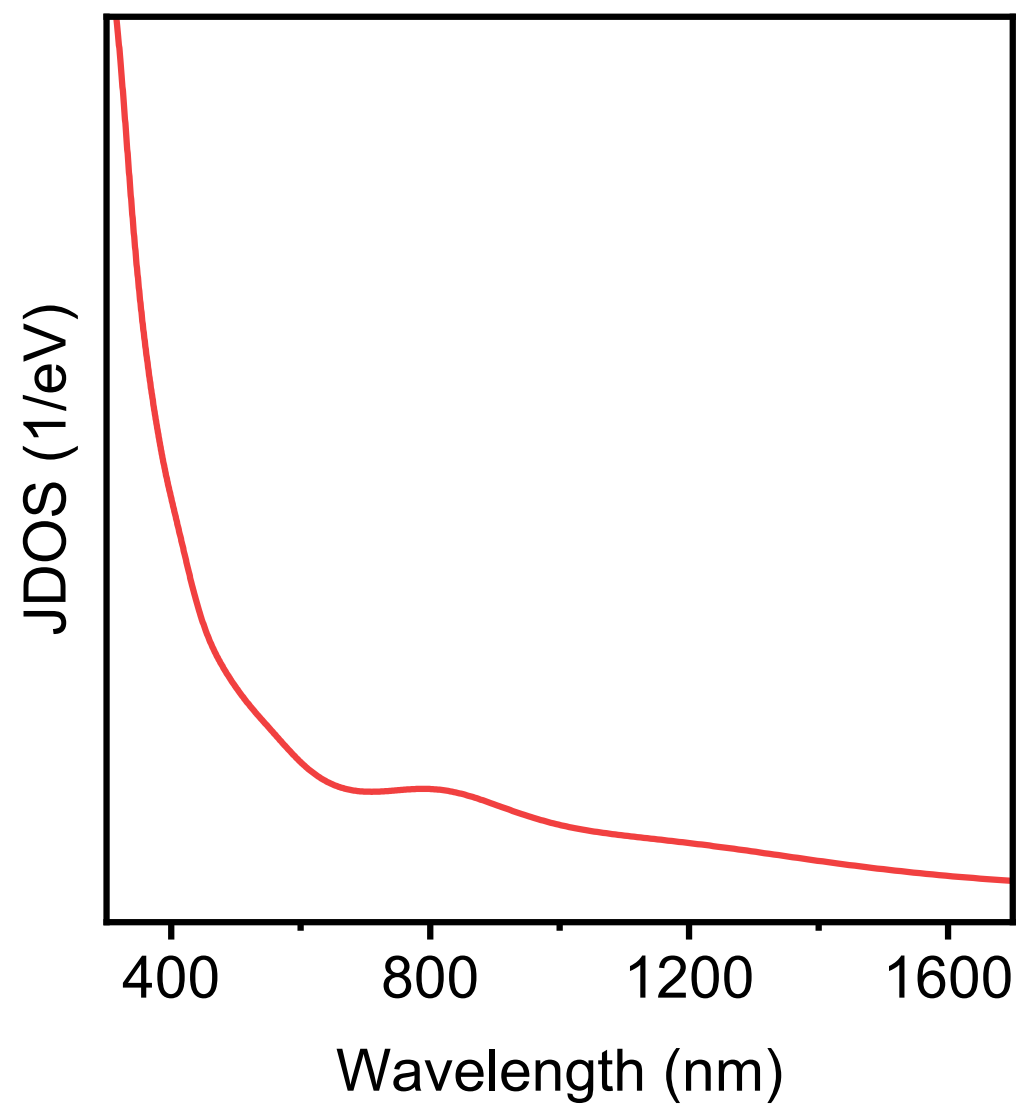

Figure S14. **DFT-calculated joint density of states (JDOS) of $Ti_3C_2Cl_2$ MXene.** JDOS of multilayer $Ti_3C_2Cl_2$ calculated with the same wavefunction for Figures S13, showing the interband transition at ~820 nm.

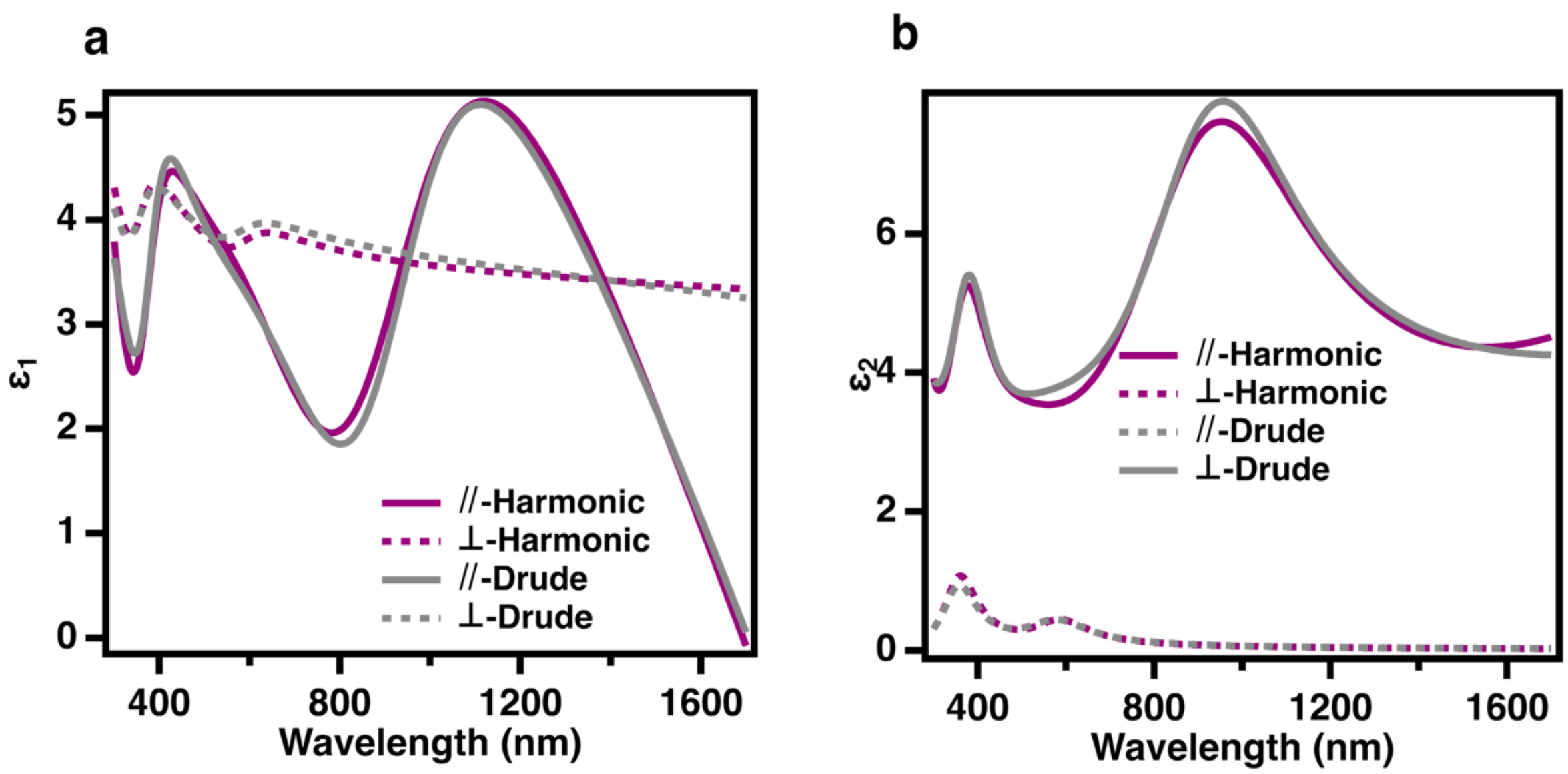

Figure S15. **Dielectric constant of $Ti_3C_2(bua)_{2/3}$ MXene from different model fittings.** (a, b) The real (ε_1) and imaginary (ε_2) parts of the dielectric constants as a function of wavelength for $Ti_3C_2(bua)_{2/3}$ MXene. // and ⊥ denote the in-plane and out-of-plane dielectric constants. The terms "Harmonic" and "Drude" denote the oscillators used to parameterize the ε_2, with the former representing a 5-harmonic oscillator model and the latter a combined model using 4 harmonic and 1 Drude oscillator. The fitting results are tabulated in Tables S3 and S4.

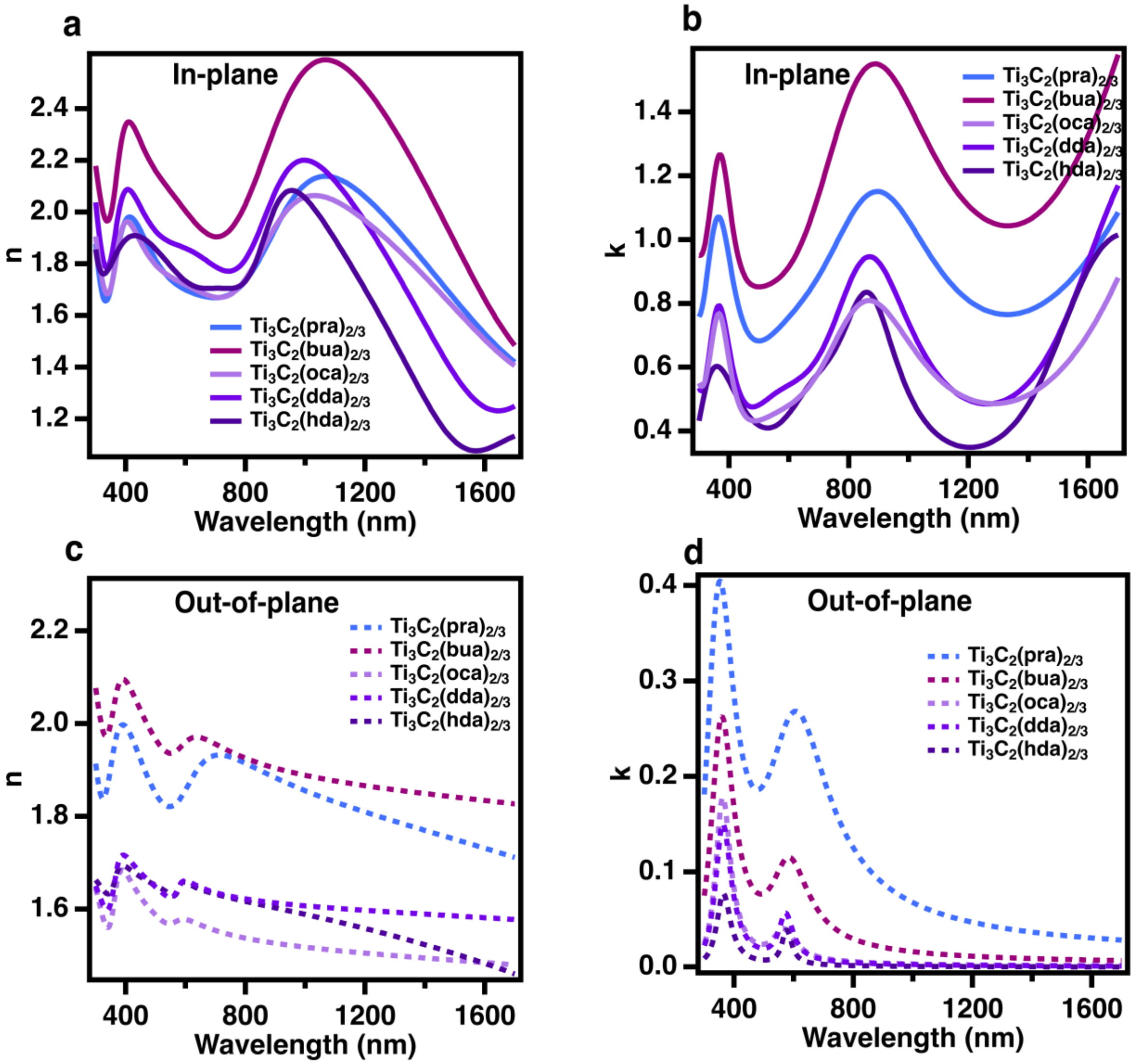

Figure S16. **Refractive index of *h*-MXenes.** (a, b) The in-plane real (*n*) and imaginary (*k*) parts of the refractive index as a function of wavelength for *h*-MXenes. (c, d) The out-of-plane real (*n*) and imaginary (*k*) parts of the refractive index as a function of wavelength for *h*-MXenes.

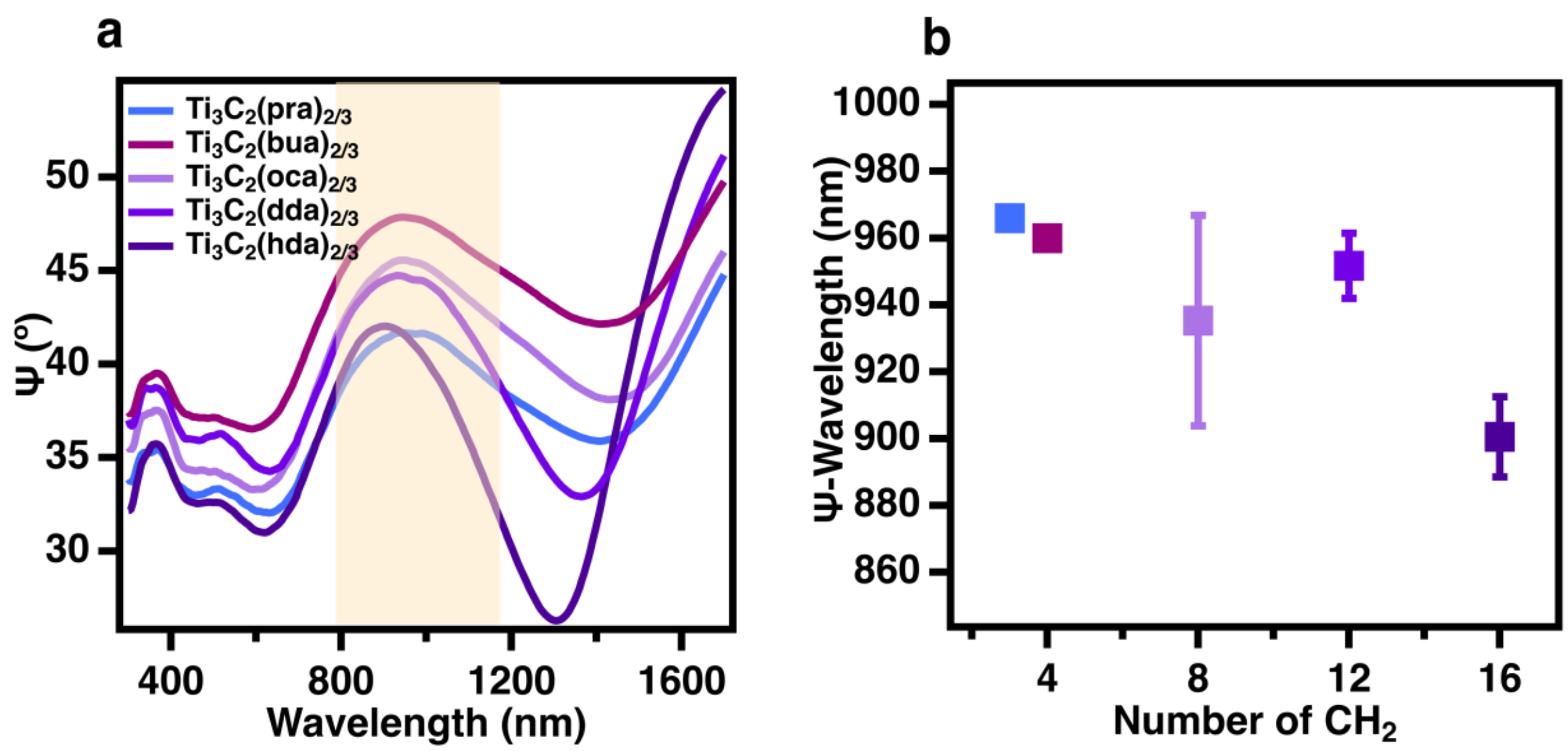

Figure S17. **Near-IR peak shift from the raw data.** (a) The measured angles Ψ as a function of wavelength at an angle of incident of 45° for *h*-MXenes under (90°, 90°, 0°) measurements. (b) Changes of the near-IR peak wavelength of Ψ (yellow highlight area in panel (a) versus the number of carbon atoms in the interlayer alkyl groups.

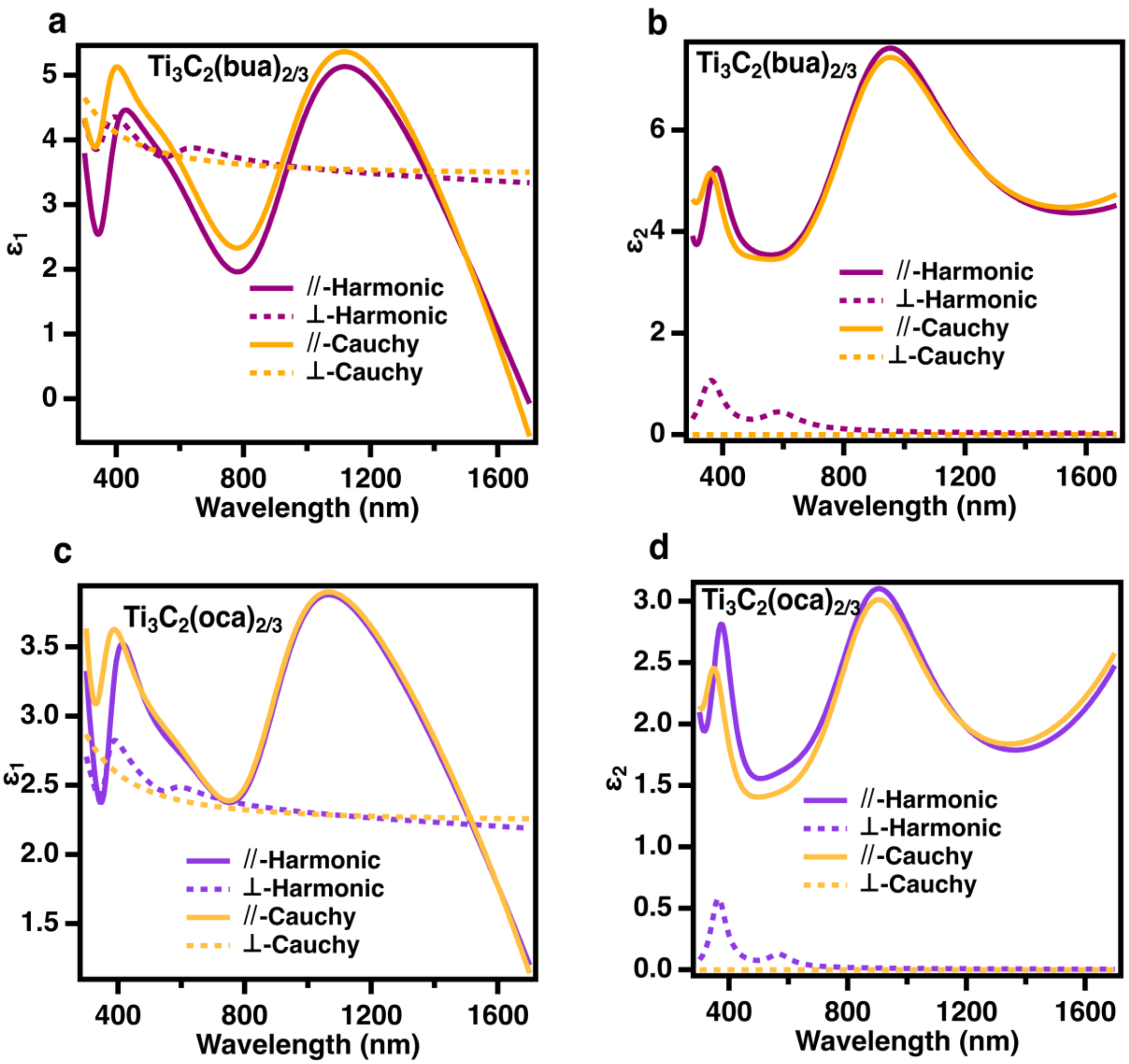

Figure S18. **Dielectric constant of $Ti_3C_2(bua)_{2/3}$ and $Ti_3C_2(oca)_{2/3}$ MXenes from different model fittings.** (a, b) The real (ε_1) and imaginary (ε_2) parts of the dielectric constants as a function of wavelength for $Ti_3C_2(bua)_{2/3}$ MXenes. // and ⊥ denote the in-plane and out-of-plane dielectric constants. The terms "Harmonic" and "Cauchy" denote the models to fit the out-of-plane dielectric constant, with the former representing harmonic oscillators to parameterize ε_2 and the latter assuming the out-of-plane is a transparent layer, whose ε_1 can be modeled by a Cauchy layer and ε_2 is zero. The fitting results are tabulated in Tables S3 and S5. (c, d) Same as (a, b), but for $Ti_3C_2(oca)_{2/3}$ MXenes. The fitting results are tabulated in Tables S6 and S7.

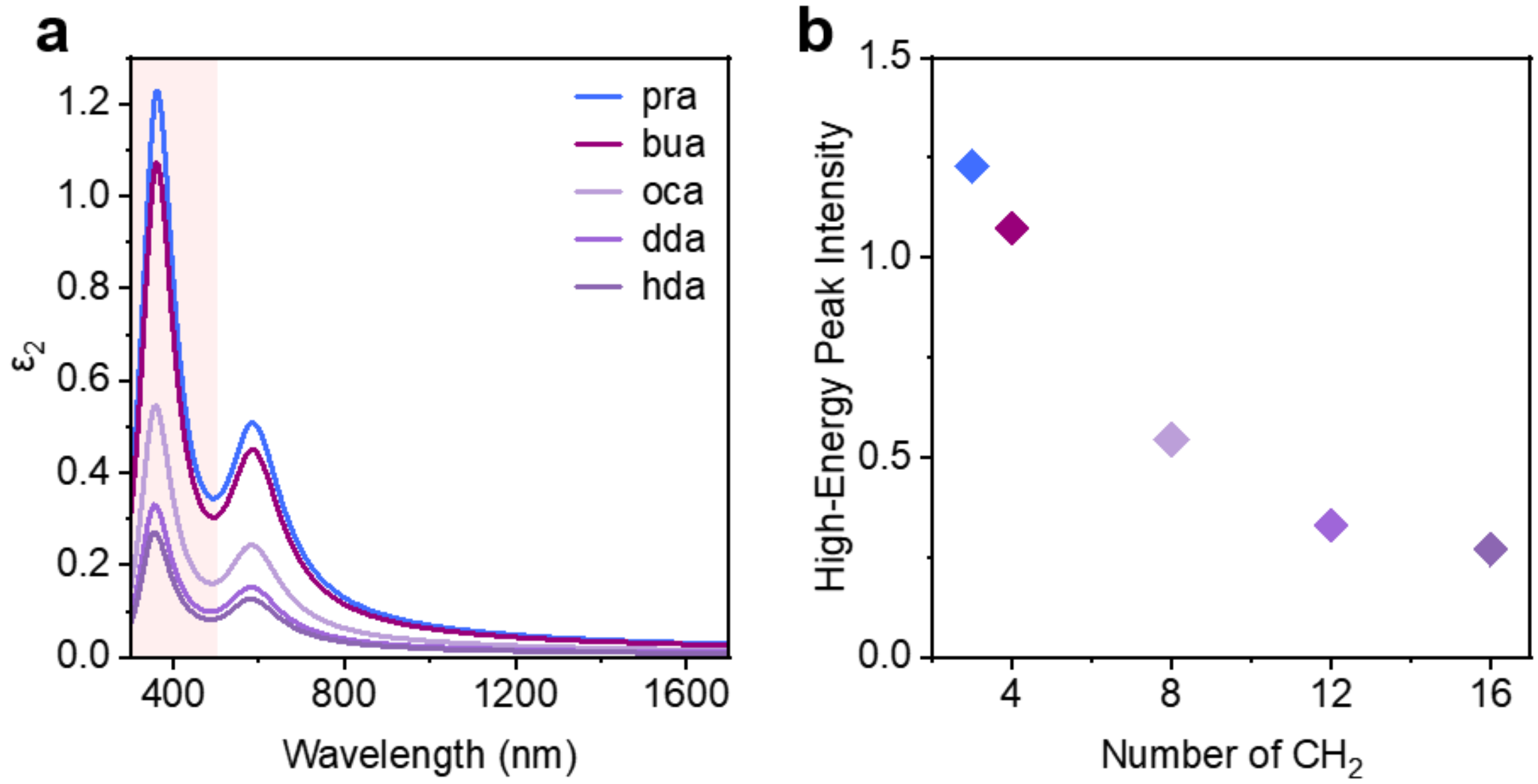

Figure S19. **Effective medium theory calculation of the out-of-plane ε_2 of *h*-MXenes.** (a) The out-of-plane imaginary (ε_2) parts of the dielectric constants for *h*-MXenes, calculated using effective medium theory. Details of the calculation are described in the Methods section. (b) Changes of the out-of-plane ε_2 high-energy peak (pink highlight area in panel a) intensity as a function of carbon atoms in the interlayer alkyl groups.

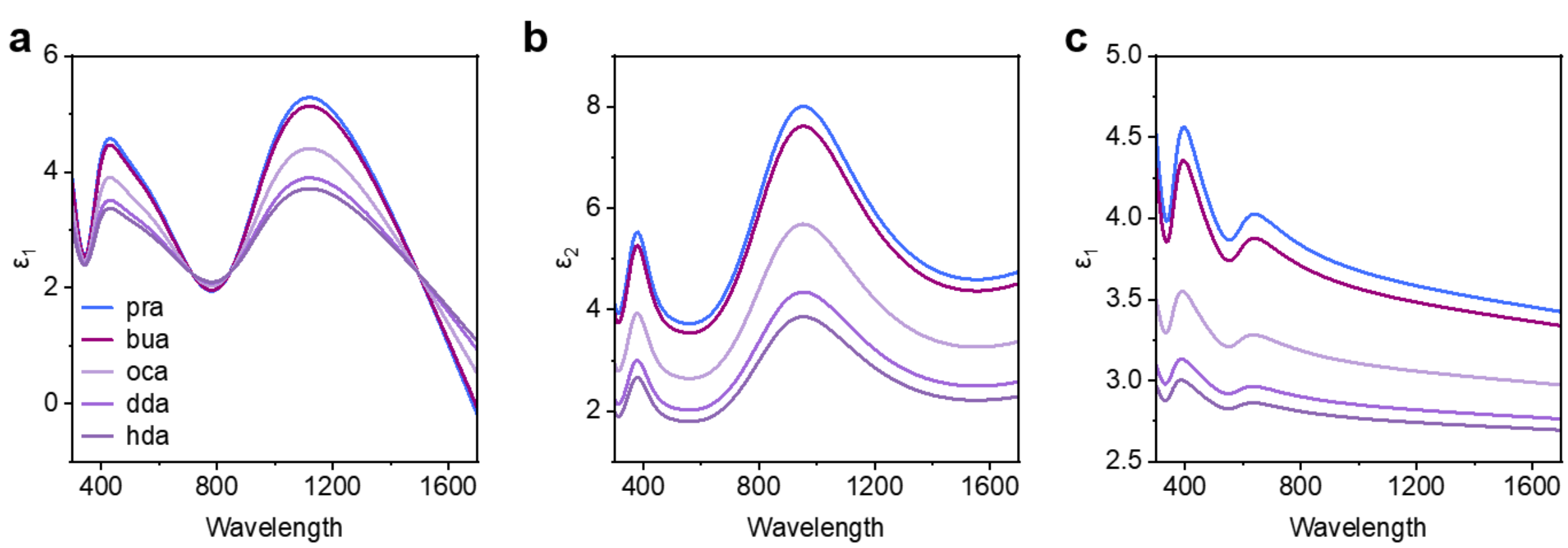

Figure S20. **Effective medium theory calculation of the *h*-MXenes.** (a,b) The in-plane real (ε_1) and imaginary (ε_2) parts of the dielectric constants for *h*-MXenes, calculated using effective medium theory. (c) The out-of-plane real (ε_1) parts of the dielectric constants for *h*-MXenes, calculated using effective medium theory. Unlike the out-of-plane ε_2 trend reproduced in Figure S19, these responses deviate from experiment, suggesting that the intrinsic optical response of the Ti_3C_2 layers may vary across the *h*-MXene series.

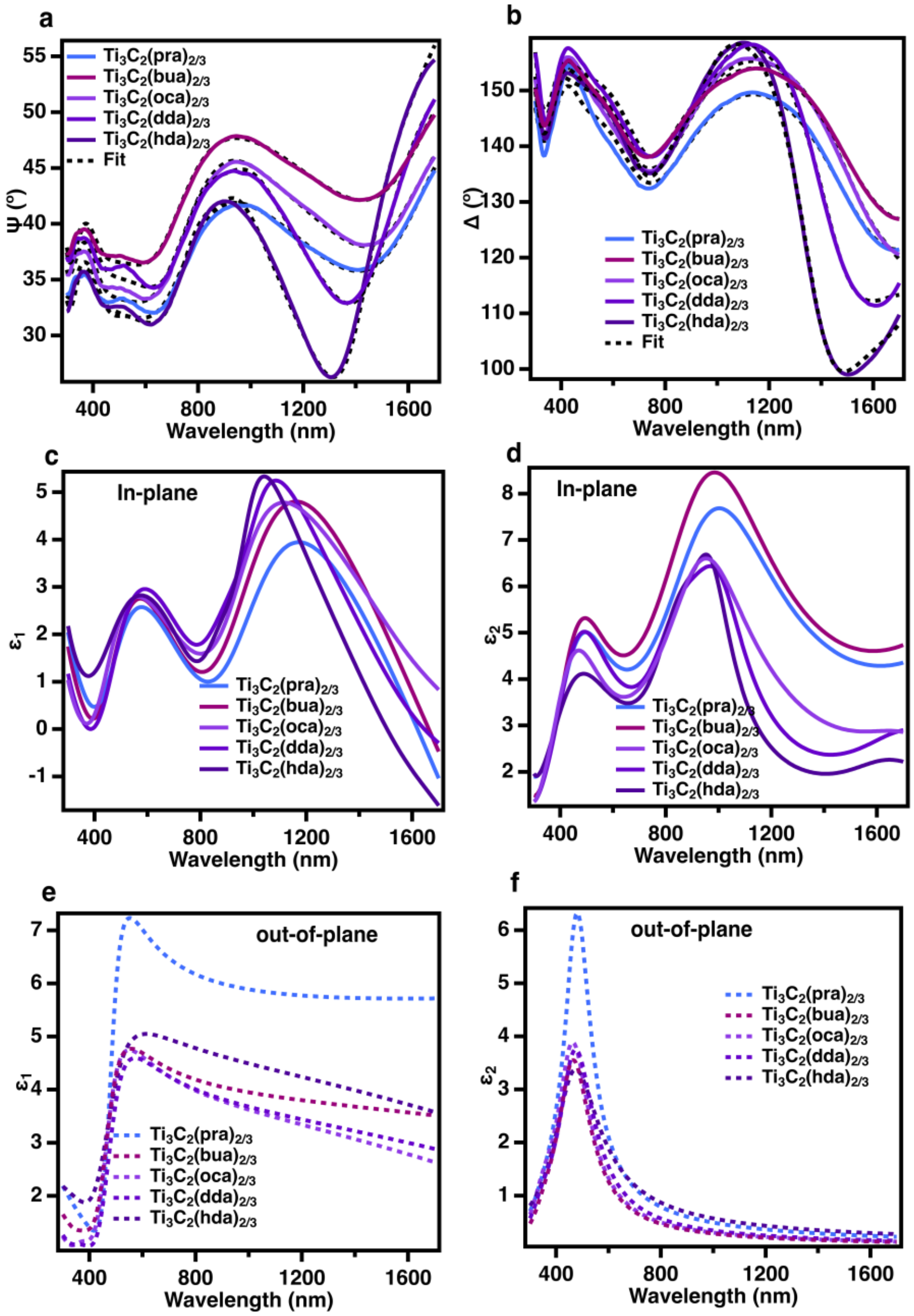

Figure S21. **Fitted ellipsometry results of *h*-MXenes under measurement (90°, 90°, 0°) only.** (a, b) Measured angles Ψ and Δ along with the best fit (dash line) as a function of wavelength at an angle of incident of 45° for *h*-MXenes. (c, d) The in-plane real (ε_1) and imaginary (ε_2) parts of the dielectric constants as a function of wavelength for *h*-MXenes. (e, f) The out-of-plane real (ε_1) and imaginary (ε_2) parts of the dielectric constants as a function of wavelength for *h*-MXenes. An anisotropic uniaxial model was used for the fitting.

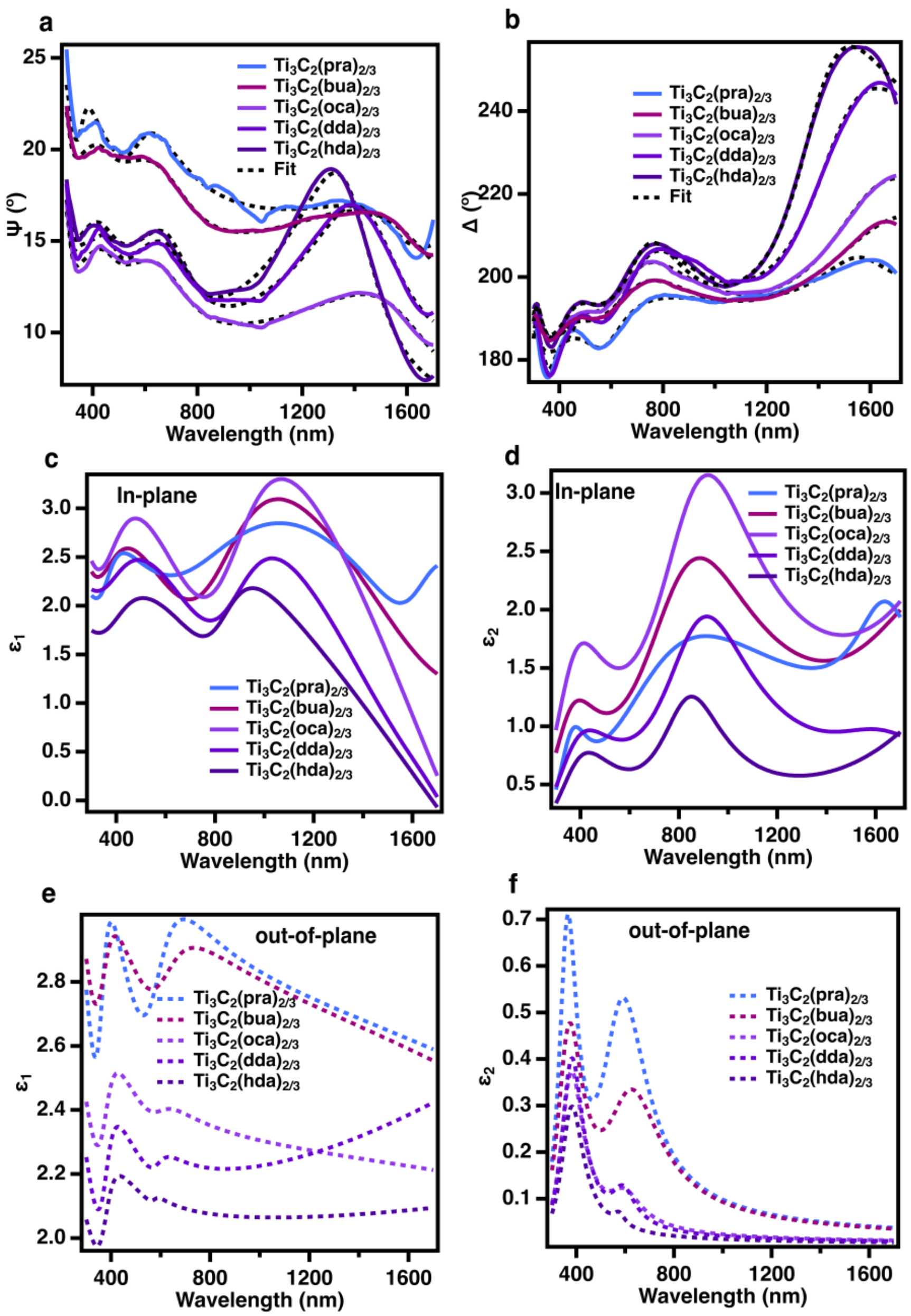

Figure S22. **Fitted ellipsometry results of *h*-MXenes under measurement (0°, 90°, 0°) only.** (a, b) Measured angles Ψ and Δ along with the best fit (dash line) as a function of wavelength at an angle of incident of 45° for *h*-MXenes. (c, d) The in-plane real (ε_1) and imaginary (ε_2) parts of the dielectric constants as a function of wavelength for *h*-MXenes. (e, f) The out-of-plane real (ε_1) and imaginary (ε_2) parts of the dielectric constants as a function of wavelength for *h*-MXenes. An anisotropic uniaxial model was used for the fitting.

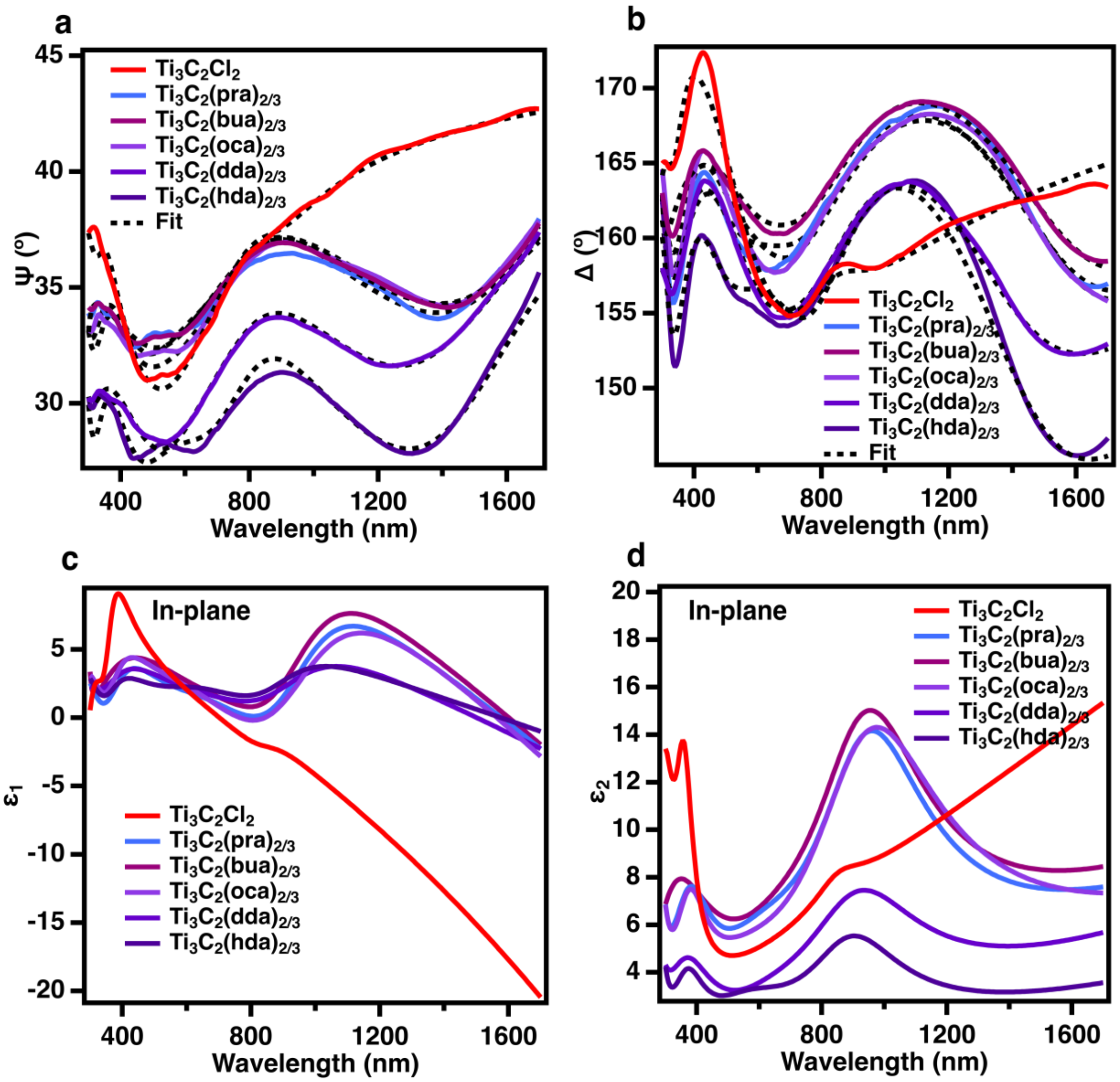

Figure S23. **Fitted ellipsometry results of $Ti_3C_2Cl_2$ and *h*-MXenes under measurement (0°, 0°, 0°) only.** (a, b) Measured angles Ψ and Δ along with the best fit (dash line) as a function of wavelength at an angle of incident of 45° for $Ti_3C_2Cl_2$ and *h*-MXenes. (c, d) The in-plane real (ε_1) and imaginary (ε_2) parts of the dielectric constants as a function of wavelength for $Ti_3C_2Cl_2$ and *h*-MXenes. An isotropic model was used for the fitting.

Table S1. **Ellipsometry global fitting results for $Ti_3C_2Cl_2$ MXene from the (90°, 90°, 0°) and (0°, 90°, 0°) measurements simultaneously.** An anisotropic uniaxial ($\varepsilon_{xx} = \varepsilon_{yy} \neq \varepsilon_{zz}$) model was used for the fitting.

MXenes		$Ti_3C_2Cl_2$	
MSE		18.14	
Uniaxial Anisotropy		In-plane	Out-of-plane
ε_1	E_∞ (eV)	0±0	0±0
	UV Pole Amplitude, A_{UV}	0±0	82±4
	UV Pole Energy, E_{UV} (eV)	11±0	6.02±0.13
	IR Pole Amplitude, A_{IR}	7.38±0.18	-0.04±0.05
ε_2 Harmonic 1	Amplitude A_n	9.36±0.26	7.08±0.58
	Breadth, Br_n (eV)	1.89±0.12	0.59±0.06
	Energy, E_n (eV)	4.42±0.04	4.79±0.03
ε_2 Harmonic 2	Amplitude A_n	8.43±0.45	2.73±0.14
	Breadth, Br_n(eV)	0.48±0.04	0.60±0.05
	Energy, E_n (eV)	3.31±0.01	3.50±0.02
ε_2 Harmonic 3	Amplitude A_n	0.80±0.05	N/A
	Breadth, Br_n(eV)	0.30±0.03	
	Energy, E_n (eV)	1.47±0.01	
ε_2 Drude	Resistivity, ρ_n (W×cm)	0.0014±0.0001	0.008±0.002
	Scattering time, τ_n (fs)	0.32±0.04	0.82±0.17

Table S2. **Ellipsometry global fitting results for $Ti_3C_2(pra)_{2/3}$ MXenes from the (90°, 90°, 0°) and (0°, 90°, 0°) measurements simultaneously.** An anisotropic uniaxial ($\varepsilon_{xx} = \varepsilon_{yy} \neq \varepsilon_{zz}$) model was used for the fitting.

MXenes		$Ti_3C_2(pra)_{2/3}$	
MSE		17.83	
Uniaxial Anisotropy		In-plane	Out-of-plane
ε_1	E_∞ (eV)	0±0	0±0
	UV Pole Amplitude, A_{UV}	0±0	100±6
	UV Pole Energy, E_{UV} (eV)	11±0	6.31±0.15
	IR Pole Amplitude, A_{IR}	1.71±0.42	0.26±0.03
ε_2 Harmonic 1	Amplitude A_n	11.85±1.38	1.51±0.07
	Breadth, Br_n (eV)	0.87±0.22	1.16±0.10
	Energy, E_n (eV)	5.18±0.20	3.49±0.03
ε_2 Harmonic 2	Amplitude A_n	2.95±0.23	0.83±0.04
	Breadth, Br_n(eV)	1.19±0.12	0.84±0.07
	Energy, E_n (eV)	3.28±0.02	2.00±0.02
ε_2 Harmonic 3	Amplitude A_n	1.42±0.31	N/A
	Breadth, Br_n(eV)	1.80±0.55	
	Energy, E_n (eV)	1.89±0.16	
ε_2 Harmonic 4	Amplitude A_n	3.24±0.42	
	Breadth, Br_n(eV)	0.69±0.06	
	Energy, E_n (eV)	1.29±0.01	
ε_2 Harmonic 5	Amplitude A_n	7.42±4.35	
	Breadth, Br_n(eV)	0.35±0.04	
	Energy, E_n (eV)	0.43±0.09	

Table S3. **Ellipsometry global fitting results for $Ti_3C_2(bua)_{2/3}$ MXene from the (90°, 90°, 0°) and (0°, 90°, 0°) measurements simultaneously.** An anisotropic uniaxial ($\varepsilon_{xx} = \varepsilon_{yy} \neq \varepsilon_{zz}$) model was used for the fitting.

MXenes		$Ti_3C_2(bua)_{2/3}$	
MSE		12.90	
Uniaxial Anisotropy		In-plane	Out-of-plane
ε_1	E_∞ (eV)	0±0	0±0
	UV Pole Amplitude, A_{UV}	0±0	150±8
	UV Pole Energy, E_{UV} (eV)	11±0	7.00±0.17
	IR Pole Amplitude, A_{IR}	2.95±0.69	0.09±0.03
ε_2 Harmonic 1	Amplitude A_n	11.11±0.84	1.06±0.06
	Breadth, Br_n (eV)	1.85±0.25	0.90±0.08
	Energy, E_n (eV)	5.59±0.11	3.44±0.03
ε_2 Harmonic 2	Amplitude A_n	3.25±0.36	0.35±0.04
	Breadth, Br_n(eV)	0.91±0.10	0.55±0.09
	Energy, E_n (eV)	3.25±0.02	2.10±0.03
ε_2 Harmonic 3	Amplitude A_n	1.63±0.23	N/A
	Breadth, Br_n(eV)	1.79±0.54	
	Energy, E_n (eV)	2.31±0.08	
ε_2 Harmonic 4	Amplitude A_n	6.25±0.23	
	Breadth, Br_n(eV)	0.79±0.03	
	Energy, E_n (eV)	1.30±0.01	
ε_2 Harmonic 5	Amplitude A_n	18.58±10.06	
	Breadth, Br_n(eV)	0.24±0.03	
	Energy, E_n (eV)	0.44±0.06	

Table S4. **Ellipsometry global fitting results for $Ti_3C_2(bua)_{2/3}$ MXene from the (90°, 90°, 0°) and (0°, 90°, 0°) measurements simultaneously.** An anisotropic uniaxial ($\varepsilon_{xx} = \varepsilon_{yy} \neq \varepsilon_{zz}$) model was used for the fitting. A Drude oscillator was used.

MXenes		$Ti_3C_2(bua)_{2/3}$	
MSE		13.80	
Uniaxial Anisotropy		In-plane	Out-of-plane
ε_1	E_∞ (eV)	0±0	0±0
	UV Pole Amplitude, A_{UV}	0±0	203±15
	UV Pole Energy, E_{UV} (eV)	11±0	7.86±0.25
	IR Pole Amplitude, A_{IR}	2.32±1.00	0.23±0.02
ε_2 Harmonic 1	Amplitude A_n	11.17±0.90	0.93±0.06
	Breadth, Br_n (eV)	1.94±0.27	0.95±0.12
	Energy, E_n (eV)	5.67±0.11	3.46±0.03
ε_2 Harmonic 2	Amplitude A_n	3.33±0.30	0.35±0.04
	Breadth, Br_n(eV)	0.96±0.10	0.62±0.10
	Energy, E_n (eV)	3.33±0.30	2.13±0.03
ε_2 Harmonic 3	Amplitude A_n	1.76±0.19	N/A
	Breadth, Br_n(eV)	1.77±0.45	
	Energy, E_n (eV)	2.18±0.06	
ε_2 Harmonic 4	Amplitude A_n	6.09±0.23	
	Breadth, Br_n(eV)	0.71±0.03	
	Energy, E_n (eV)	1.30±0.01	
ε_2 Drude	Resistivity, ρ_n (W×cm)	0.0035±0.0006	
	Scattering time, τ_n (fs)	0.41±0.26	

Table S5. **Ellipsometry global fitting results for $Ti_3C_2(bua)_{2/3}$ MXene from the (90°, 90°, 0°) and (0°, 90°, 0°) measurements simultaneously.** An anisotropic uniaxial ($\varepsilon_{xx} = \varepsilon_{yy} \neq \varepsilon_{zz}$) model was used for the fitting. The out-of-plane was assumed to be a Cauchy transparent layer.

MXenes		$Ti_3C_2(bua)_{2/3}$			
MSE		19.00			
Uniaxial Anisotropy		In-plane	Out-of-plane		
ε_1	E_∞ (eV)	0±0	ε_1 Cauchy	A	1.86±0.01
	UV Pole Amplitude, A_{UV}	0±0		B	0.03±0.01
	UV Pole Energy, E_{UV} (eV)	11±0		C	0±0
	IR Pole Amplitude, A_{IR}	0.48±0.54			
ε_2 Harmonic 1	Amplitude A_n	9.94±1.77	ε_2	0	
	Breadth, Br_n (eV)	2.98±1.03			
	Energy, E_n (eV)	6.00±0.22			
ε_2 Harmonic 2	Amplitude A_n	2.39±0.51			
	Breadth, Br_n(eV)	0.99±0.22			
	Energy, E_n (eV)	3.40±0.03			
ε_2 Harmonic 3	Amplitude A_n	1.20±0.23			
	Breadth, Br_n(eV)	1.42±0.36			
	Energy, E_n (eV)	2.36±0.07			
ε_2 Harmonic 4	Amplitude A_n	6.31±0.16			
	Breadth, Br_n(eV)	0.79±0.03			
	Energy, E_n (eV)	1.30±0.01			
ε_2 Harmonic 5	Amplitude A_n	102.13±16.00			
	Breadth, Br_n(eV)	0.15±0.07			
	Energy, E_n (eV)	0.31±0.11			

Table S6. **Ellipsometry global fitting results for $Ti_3C_2(oca)_{2/3}$ MXene from the** (90°, 90°, 0°) and (0°, 90°, 0°) **measurements simultaneously.** An anisotropic uniaxial ($\varepsilon_{xx} = \varepsilon_{yy} \neq \varepsilon_{zz}$) model was used for the fitting.

MXenes		$Ti_3C_2(oca)_{2/3}$	
MSE		18.21	
Uniaxial Anisotropy		In-plane	Out-of-plane
ε_1	E_∞ (eV)	1.11±0.24	0±0
	UV Pole Amplitude, A_{UV}	0±0	143±14
	UV Pole Energy, E_{UV} (eV)	11±0	8.15±0.35
	IR Pole Amplitude, A_{IR}	0.23±0.35	0.05±0.02
ε_2 Harmonic 1	Amplitude A_n	7.14±0.78	0.58±0.05
	Breadth, Br_n (eV)	1.04±0.25	0.59±0.09
	Energy, E_n (eV)	5.12±0.20	3.41±0.02
ε_2 Harmonic 2	Amplitude A_n	1.98±0.15	0.10±0.03
	Breadth, Br_n(eV)	0.87±0.11	0.35±0.12
	Energy, E_n (eV)	3.31±0.02	2.19±0.04
ε_2 Harmonic 3	Amplitude A_n	0.86±0.11	N/A
	Breadth, Br_n(eV)	1.68±0.49	
	Energy, E_n (eV)	2.13±0.07	
ε_2 Harmonic 4	Amplitude A_n	2.41±0.16	
	Breadth, Br_n(eV)	0.68±0.06	
	Energy, E_n (eV)	1.36±0.01	
ε_2 Harmonic 5	Amplitude A_n	13.68±4.56	
	Breadth, Br_n(eV)	0.22±0.02	
	Energy, E_n (eV)	0.46±0.03	

Table S7. **Ellipsometry global fitting results for $Ti_3C_2(oca)_{2/3}$ MXene from the (90°, 90°, 0°) and (0°, 90°, 0°) measurements simultaneously.** An anisotropic uniaxial ($\varepsilon_{xx} = \varepsilon_{yy} \neq \varepsilon_{zz}$) model was used for the fitting. The out-of-plane was assumed to be a Cauchy transparent layer.

MXenes		$Ti_3C_2(oca)_{2/3}$			
MSE		19.86			
Uniaxial Anisotropy		In-plane	Out-of-plane		
ε_1	E_∞ (eV)	1.05±0.36	ε_1 Cauchy	A	1.50±0.01
	UV Pole Amplitude, A_{UV}	0±0		B	0.02±0.01
	UV Pole Energy, E_{UV} (eV)	11±0		C	0±0
	IR Pole Amplitude, A_{IR}	-0.87±0.56			
ε_2 Harmonic 1	Amplitude A_n	9.82±2.41	e2	0	
	Breadth, Br_n (eV)	0.78±0.41			
	Energy, E_n (eV)	5.15±0.30			
ε_2 Harmonic 2	Amplitude A_n	1.66±0.30			
	Breadth, Br_n(eV)	1.12±0.20			
	Energy, E_n (eV)	3.52±0.04			
ε_2 Harmonic 3	Amplitude A_n	0.74±0.11			
	Breadth, Br_n(eV)	1.86±0.58			
	Energy, E_n (eV)	2.22±0.07			
ε_2 Harmonic 4	Amplitude A_n	2.37±0.13			
	Breadth, Br_n(eV)	0.69±0.03			
	Energy, E_n (eV)	1.37±0.01			
ε_2 Harmonic 5	Amplitude A_n	32.29±13.20			
	Breadth, Br_n(eV)	0.17±0.02			
	Energy, E_n (eV)	0.40±0.03			

Table S8. **Ellipsometry global fitting results for $Ti_3C_2(dda)_{2/3}$ MXene from the (90°, 90°, 0°) and (0°, 90°, 0°) measurements simultaneously.** An anisotropic uniaxial ($\varepsilon_{xx} = \varepsilon_{yy} \neq \varepsilon_{zz}$) model was used for the fitting.

MXenes		$Ti_3C_2(dda)_{2/3}$	
MSE		47.95	
Uniaxial Anisotropy		In-plane	Out-of-plane
ε_1	E_∞ (eV)	2.14±0.49	0±0
	UV Pole Amplitude, A_{UV}	0±0	276±122
	UV Pole Energy, E_{UV} (eV)	11±0	10.61±2.19
	IR Pole Amplitude, A_{IR}	2.61±0.25	0.05±0.05
ε_2 Harmonic 1	Amplitude A_n	7.50±2.68	0.50±0.14
	Breadth, Br_n (eV)	0.68±0.48	0.61±0.24
	Energy, E_n (eV)	4.84±0.37	3.41±0.08
ε_2 Harmonic 2	Amplitude A_n	2.41±0.37	0.16±0.08
	Breadth, Br_n(eV)	0.99±0.30	0.22±0.17
	Energy, E_n (eV)	3.31±0.06	2.16±0.05
ε_2 Harmonic 3	Amplitude A_n	1.26±0.25	N/A
	Breadth, Br_n(eV)	1.50±0.74	
	Energy, E_n (eV)	2.08±0.11	
ε_2 Harmonic 4	Amplitude A_n	3.04±0.35	
	Breadth, Br_n(eV)	0.55±0.06	
	Energy, E_n (eV)	1.37±0.01	
ε_2 Harmonic 5	Amplitude A_n	3.22±0.74	
	Breadth, Br_n(eV)	0.23±0.02	
	Energy, E_n (eV)	0.65±0.03	

Table S9. **Ellipsometry global fitting results for $Ti_3C_2(hda)_{2/3}$ MXene from the (90°, 90°, 0°) and (0°, 90°, 0°) measurements simultaneously.** An anisotropic uniaxial ($\varepsilon_{xx} = \varepsilon_{yy} \neq \varepsilon_{zz}$) model was used for the fitting.

MXenes		$Ti_3C_2(hda)_{2/3}$	
MSE		52.77	
Uniaxial Anisotropy		In-plane	Out-of-plane
ε_1	E_∞ (eV)	1.86±0.65	0±0
	UV Pole Amplitude, A_{UV}	0±0	407±242
	UV Pole Energy, E_{UV} (eV)	11±0	12.48±3.56
	IR Pole Amplitude, A_{IR}	2.54±0.13	0.29±0.05
ε_2 Harmonic 1	Amplitude A_n	8.80±1.32	0.31±0.11
	Breadth, Br_n (eV)	0.48±0.10	0.45±0.21
	Energy, E_n (eV)	5.08±0.85	3.20±0.08
ε_2 Harmonic 2	Amplitude A_n	2.03±0.37	0.08±0.08
	Breadth, Br_n(eV)	2.40±0.77	0.18±0.17
	Energy, E_n (eV)	3.23±0.20	1.96±0.10
ε_2 Harmonic 3	Amplitude A_n	1.09±0.54	N/A
	Breadth, Br_n(eV)	0.67±0.27	
	Energy, E_n (eV)	1.64±0.12	
ε_2 Harmonic 4	Amplitude A_n	1.98±0.59	
	Breadth, Br_n(eV)	0.34±0.08	
	Energy, E_n (eV)	1.38±0.02	
ε_2 Harmonic 5	Amplitude A_n	1.96±0.20	
	Breadth, Br_n(eV)	0.24±0.02	
	Energy, E_n (eV)	0.71±0.01	

Supporting References

(1) Anayee, M.; Wang, R. (John); Downes, M.; Ippolito, S.; Gogotsi, Y. Layer-by-Layer Mechanism of the MAX Phase to MXene Transformation. *Matter* **2025**, *8* (6). https://doi.org/10.1016/j.matt.2025.102092.

(2) Kamysbayev, V.; Filatov, A. S.; Hu, H.; Rui, X.; Lagunas, F.; Wang, D.; Klie, R. F.; Talapin, D. V. Covalent Surface Modifications and Superconductivity of Two-Dimensional Metal Carbide MXenes. *Science* **2020**, *369* (6506), 979–983. https://doi.org/10.1126/science.aba8311.

(3) Zhou, C.; Wang, D.; Lagunas, F.; Atterberry, B.; Lei, M.; Hu, H.; Zhou, Z.; Filatov, A. S.; Jiang, D.; Rossini, A. J.; Klie, R. F.; Talapin, D. V. Hybrid Organic–Inorganic Two-Dimensional Metal Carbide MXenes with Amido- and Imido-Terminated Surfaces. *Nat. Chem.* **2023**, *15* (12), 1722–1729. https://doi.org/10.1038/s41557-023-01288-w.

(4) Shamsabadi, A. A.; Fang, H.; Zhang, D.; Thakur, A.; Chen, C. Y.; Zhang, A.; Wang, H.; Anasori, B.; Soroush, M.; Gogotsi, Y.; Fakhraai, Z. The Evolution of MXenes Conductivity and Optical Properties Upon Heating in Air. *Small Methods* **2023**, *7* (10), 2300568. https://doi.org/10.1002/smtd.202300568.

(5) Fang, H.; Fang, Z.; Thakur, A.; Rad, V.; Chandran Balachandran Sajitha, N.; Michałowski, P. P.; Soroush, M.; Anasori, B.; Rappe, A. M.; Fakhraai, Z. Band-like Optical Signatures of $Ti_3C_2T_x$ MXenes. *J. Phys. Chem. C* **2026**, *130* (6), 2294–2306. https://doi.org/10.1021/acs.jpcc.5c08501.

(6) Fang, H.; Thakur, A.; Zahmatkeshsaredorahi, A.; Fang, Z.; Rad, V.; Shamsabadi, A. A.; Pereyra, C.; Soroush, M.; Rappe, A. M.; Xu, X. G.; Anasori, B.; Fakhraai, Z. Stabilizing $Ti_3C_2T_x$ MXene Flakes in Air by Removing Confined Water. *Proceedings of the National Academy of Sciences* **2024**, *121* (28), e2400084121. https://doi.org/10.1073/pnas.2400084121.

(7) Fang, H.; Fang, Z.; Thakur, A.; Anasori, B.; Rappe, A. M.; Fakhraai, Z. Different Charge Transport Mechanisms in $Ti_3C_2T_x$ MXene Monoflakes and Multiflakes. *J. Phys. Chem. Lett.* **2025**, *16* (30), 7515–7521. https://doi.org/10.1021/acs.jpclett.5c01439.

(8) Ernst, R. R.; Anderson, W. A. Application of Fourier Transform Spectroscopy to Magnetic Resonance. *Rev. Sci. Instrum.* **1966**, *37* (1), 93–102. https://doi.org/10.1063/1.1719961.

(9) Ishii, Y.; Tycko, R. Sensitivity Enhancement in Solid State 15N NMR by Indirect Detection with High-Speed Magic Angle Spinning. *Journal of Magnetic Resonance* **2000**, *142* (1), 199–204. https://doi.org/10.1006/jmre.1999.1976.

(10) Wiench, J. W.; Bronnimann, C. E.; Lin, V. S.-Y.; Pruski, M. Chemical Shift Correlation NMR Spectroscopy with Indirect Detection in Fast Rotating Solids: Studies of Organically Functionalized Mesoporous Silicas. *J. Am. Chem. Soc.* **2007**, *129* (40), 12076–12077. https://doi.org/10.1021/ja074746+.

(11) Marion, D.; Bax, A. Baseline Correction of 2D FT NMR Spectra Using a Simple Linear Prediction Extrapolation of the Time-Domain Data. *Journal of Magnetic Resonance (1969)* **1989**, *83* (1), 205–211. https://doi.org/10.1016/0022-2364(89)90307-7.

(12) Metz, G.; Wu, X. L.; Smith, S. O. Ramped-Amplitude Cross Polarization in Magic-Angle-Spinning NMR. *Journal of Magnetic Resonance, Series A* **1994**, *110* (2), 219–227. https://doi.org/10.1006/jmra.1994.1208.

(13) Larsen, F. H.; Jakobsen, H. J.; Ellis, P. D.; Nielsen, N. Chr. QCPMG-MAS NMR of Half-Integer Quadrupolar Nuclei. *Journal of Magnetic Resonance* **1998**, *131* (1), 144–147. https://doi.org/10.1006/jmre.1997.1341.

(14) Harris, R. K.; Becker, E. D.; Cabral de Menezes, S. M.; Goodfellow, R.; Granger, P. NMR Nomenclature: Nuclear Spin Properties and Conventions for Chemical Shifts: IUPAC Recommendations 2001. *Solid State Nuclear Magnetic Resonance* **2002**, *22* (4), 458–483. https://doi.org/10.1006/snmr.2002.0063.

(15) Giannozzi, P.; Baroni, S.; Bonini, N.; Calandra, M.; Car, R.; Cavazzoni, C.; Ceresoli, D.; Chiarotti, G. L.; Cococcioni, M.; Dabo, I.; Dal Corso, A.; de Gironcoli, S.; Fabris, S.; Fratesi, G.; Gebauer, R.; Gerstmann, U.; Gougoussis, C.; Kokalj, A.; Lazzeri, M.; Martin-Samos, L.; Marzari, N.; Mauri, F.; Mazzarello, R.; Paolini, S.; Pasquarello, A.; Paulatto, L.; Sbraccia, C.; Scandolo, S.; Sclauzero, G.; Seitsonen, A. P.; Smogunov, A.; Umari, P.; Wentzcovitch, R. M. QUANTUM ESPRESSO: A Modular and Open-Source Software Project for Quantum Simulations of Materials. *J. Phys.: Condens. Matter* **2009**, *21* (39), 395502. https://doi.org/10.1088/0953-8984/21/39/395502.

(16) Giannozzi, P.; Andreussi, O.; Brumme, T.; Bunau, O.; Buongiorno Nardelli, M.; Calandra, M.; Car, R.; Cavazzoni, C.; Ceresoli, D.; Cococcioni, M.; Colonna, N.; Carnimeo, I.; Dal Corso, A.; de Gironcoli, S.; Delugas, P.; DiStasio, R. A.; Ferretti, A.; Floris, A.; Fratesi, G.; Fugallo, G.; Gebauer, R.; Gerstmann, U.; Giustino, F.; Gorni, T.; Jia, J.; Kawamura, M.; Ko, H.-Y.; Kokalj, A.; Küçükbenli, E.; Lazzeri, M.; Marsili, M.; Marzari, N.; Mauri, F.; Nguyen, N. L.; Nguyen, H.-V.; Otero-de-la-Roza, A.; Paulatto, L.; Poncé, S.; Rocca, D.; Sabatini, R.; Santra, B.; Schlipf, M.; Seitsonen, A. P.; Smogunov, A.; Timrov, I.; Thonhauser, T.; Umari, P.; Vast, N.; Wu, X.; Baroni, S. Advanced Capabilities for Materials Modelling with Quantum ESPRESSO. *J. Phys.: Condens. Matter* **2017**, *29* (46), 465901. https://doi.org/10.1088/1361-648X/aa8f79.

(17) Giannozzi, P.; Baseggio, O.; Bonfà, P.; Brunato, D.; Car, R.; Carnimeo, I.; Cavazzoni, C.; de Gironcoli, S.; Delugas, P.; Ferrari Ruffino, F.; Ferretti, A.; Marzari, N.; Timrov, I.; Urru, A.; Baroni, S. Quantum ESPRESSO toward the Exascale. *The Journal of Chemical Physics* **2020**, *152* (15), 154105. https://doi.org/10.1063/5.0005082.

(18) Wellendorff, J.; Lundgaard, K. T.; Møgelhøj, A.; Petzold, V.; Landis, D. D.; Nørskov, J. K.; Bligaard, T.; Jacobsen, K. W. Density Functionals for Surface Science: Exchange-Correlation Model Development with Bayesian Error Estimation. *Phys. Rev. B* **2012**, *85* (23), 235149. https://doi.org/10.1103/PhysRevB.85.235149.

(19) Hamann, D. R. Optimized Norm-Conserving Vanderbilt Pseudopotentials. *Phys. Rev. B* **2013**, *88* (8), 085117. https://doi.org/10.1103/PhysRevB.88.085117.

(20) Schlipf, M.; Gygi, F. Optimization Algorithm for the Generation of ONCV Pseudopotentials. *Computer Physics Communications* **2015**, *196*, 36–44. https://doi.org/10.1016/j.cpc.2015.05.011.

(21) DeCrescent, R. A.; Venkatesan, N. R.; Dahlman, C. J.; Kennard, R. M.; Chabinyc, M. L.; Schuller, J. A. Optical Constants and Effective-Medium Origins of Large Optical Anisotropies in Layered Hybrid Organic/Inorganic Perovskites. *ACS Nano* **2019**, *13* (9), 10745–10753. https://doi.org/10.1021/acsnano.9b05504.

(22) Poddubny, A.; Iorsh, I.; Belov, P.; Kivshar, Y. Hyperbolic Metamaterials. *Nature Photon* **2013**, *7* (12), 948–957. https://doi.org/10.1038/nphoton.2013.243.

(23) Marinopoulos, A. G.; Reining, L.; Olevano, V.; Rubio, A.; Pichler, T.; Liu, X.; Knupfer, M.; Fink, J. Anisotropy and Interplane Interactions in the Dielectric Response of Graphite. *Phys. Rev. Lett.* **2002**, *89* (7), 076402. https://doi.org/10.1103/PhysRevLett.89.076402.

(24) Marinopoulos, A. G.; Reining, L.; Rubio, A.; Olevano, V. Ab Initio Study of the Optical Absorption and Wave-Vector-Dependent Dielectric Response of Graphite. *Phys. Rev. B* **2004**, *69* (24), 245419. https://doi.org/10.1103/PhysRevB.69.245419.
(25) Johari, P.; Shenoy, V. B. Tunable Dielectric Properties of Transition Metal Dichalcogenides. *ACS Nano* **2011**, *5* (7), 5903–5908. https://doi.org/10.1021/nn201698t.
(26) Cudazzo, P.; Ruotsalainen, K. O.; Sahle, C. J.; Al-Zein, A.; Berger, H.; Navarro-Moratalla, E.; Huotari, S.; Gatti, M.; Rubio, A. High-Energy Collective Electronic Excitations in Layered Transition-Metal Dichalcogenides. *Phys. Rev. B* **2014**, *90* (12), 125125. https://doi.org/10.1103/PhysRevB.90.125125.
(27) Cudazzo, P.; Gatti, M.; Rubio, A. Local-Field Effects on the Plasmon Dispersion of Two-Dimensional Transition Metal Dichalcogenides. *New J. Phys.* **2013**, *15* (12), 125005. https://doi.org/10.1088/1367-2630/15/12/125005.
(28) Tancogne-Dejean, N.; Giorgetti, C.; Véniard, V. Optical Properties of Surfaces with Supercell Ab Initio Calculations: Local-Field Effects. *Phys. Rev. B* **2015**, *92* (24), 245308. https://doi.org/10.1103/PhysRevB.92.245308.
(29) Lončarić, I.; Rukelj, Z.; Silkin, V. M.; Despoja, V. Strong Two-Dimensional Plasmon in Li-Intercalated Hexagonal Boron-Nitride Film with Low Damping. *npj 2D Mater Appl* **2018**, *2* (1), 33. https://doi.org/10.1038/s41699-018-0078-y.
(30) Woollam, J. CompleteEASE software manual (version 6.54). *Lincoln, USA: JA Woollam Co* **2020**.
